# Archeochemistry reveals the first steps into modern industrial brewing

**DOI:** 10.1038/s41598-022-12943-6

**Published:** 2022-06-03

**Authors:** Stefan A. Pieczonka, Martin Zarnkow, Philippe Diederich, Mathias Hutzler, Nadine Weber, Fritz Jacob, Michael Rychlik, Philippe Schmitt-Kopplin

**Affiliations:** 1 Chair of Analytical Food Chemistry, Technical University of Munich, Maximus-von-Imhof-Forum 2, 85354 Freising, Germany; 2 Research Unit Analytical BioGeoChemistry, Helmholtz Munich, Ingolstädter Landstraße 1, 85764 Neuherberg, Germany; 3 Research Center Weihenstephan for Brewing and Food Quality, Technical University of Munich, Alte Akademie 3, 85354 Freising, Germany

**Keywords:** Metabolomics, Analytical chemistry, Communicating chemistry

## Abstract

A historical beer, dated to the German Empire era, was recently found in northern Germany. Its chemical composition represents a unique source of insights into brewing culture of the late nineteenth century when pioneer innovations laid the foundations for industrial brewing. Complementary analytics including metabolomics, microbiological, sensory, and beer attribute analysis revealed its molecular profile and certify the unprecedented good storage condition even after 130 years in the bottle. Comparing its chemical signature to that of four hundred modern brews allowed to describe molecular fingerprints teaching us about technological aspects of historical beer brewing. Several critical production steps such as malting and germ treatment, wort preparation and fermentation, filtration and storage, and compliance with the Bavarian Purity Law left detectable molecular imprints. In addition, the aging process of the drinkable brew could be analyzed on a chemical level and resulted in an unseen diversity of hops- and Maillard-derived compounds. Using this archeochemical forensic approach, the historical production process of a culturally significant beverage could be traced and the ravages of time made visible.

## Introduction

The birth and social evolution of humans and civilizations is closely related to the cultural heritage of beer production^1,2^. The reason for settling down may have been feasting, as suggested in the 11,000 thousand-year-old excavation site of Göbekli Tepe^3^. The latest excavations from Abydos, which revealed the oldest known mass-production brewery (over 20,000 L a batch) dated at around 3,000 BC, again highlighted the importance of beer as food and ritual addition in early civilizations^4^. Brewing goes hand in hand with fundamental changes in human culture and jurisprudence. As one of the oldest fermented beverages of ancient origin^5^, the historical meaning of brewing lies in the cultural transition towards producing durable beverages from domesticated grain cultivation. To ensure the quality and bacteriostatic property of beer, the Bavarian Purity Law (1516)^6^ was established as one of the most significant food legislations of the early modern period. The transition to modern industrial brewing in the late nineteenth century was made possible by inventions strongly linked to beer and brewing research.

The fascination of fermentation processes, which was puzzling until the early modern era, fostered and was a driving force for innovation and science. The discovery of aerobic and anaerobic metabolic pathways^7^ and the principle of pasteurization^8^, thus the concept of modern food hygiene, are closely linked to beer and yeast research. The isolation of individual yeast cells and cultured yeasts^9^, as well as the first “refrigeration apparatus” by Linde^10^ for brewing bottom-fermented beer are significant achievements for today’s advancing civilization.

Rising from such pioneer works, the field of analytical chemistry nowadays is implemented to characterize the organic and inorganic residues of ancient and historical finds. In the recent past, archeochemistry evolved from the analysis of single marker compounds like tartaric acid (indication of winemaking)^11,12^, oxalic acid (indication of brewing)^5^ or acetic acid/lactic acid (indication of spoilage after fermentation)^13^ to a more holistic approach integrating multiple analytical fields and metabolomics. Walther et al.^14^ sequenced the genome of the oldest pure culture yeast strain *Saccharomyces carlsbergensis* (1883), thereby specifying their ploidy and genetic evolution, and detected it in beer samples presumably from the 1880s to 1900s^15^. Beer bottles found in a shipwreck in the Baltic Sea dated to the 1840s^16^ were analyzed by means of reversed-phase and ion exchange LC and GC targeted approaches revealing insights in their hops and aroma compounds, despite contamination. GC-olfactometry and sensory analysis gave insights into the complex aroma profile of the Shackleton’s whisky (late nineteenth century)^17^. Comprehensive non-targeted approaches utilizing the mass resolution and accuracy of high-field Fourier-transform ion cyclotron mass spectrometry (FTICR-MS) and Nuclear Magnetic resonance spectroscopy (NMR) were carried out investigating a champagne dated to the 1840s^18^ and an unidentified wine sample from the late eighteenth to nineteenth century^19^. Based on the resolved molecular composition, the story of historical winemaking and champagne production could be traced step by step^18^ in comparing modern and historical references. In addition to beer attribute, sensory and microbiological investigations, we adapted here such a comprehensive concept for the FTICR-MS and NMR-based characterization of a historical and well-preserved beer sample from the late nineteenth century. The resolved metabolic profile and its chemical transformation during storage, including thousands of yet-unknown structures (“dark metabolome”), provides a molecular insight into the historical beer composition. Comprehensive non-targeted archeochemistry, even after more than a 100 years, allows conclusions with regard to the industrial brewing revolution at that time.

## Results and discussion

### Discovery, beer attributes and sensory characterization

A newspaper article from June 19th (Supplementary Figure S1), 1978 refers to an extraordinary find: corked, wired and sealed, a bottle was found during the clearing up of a commercial building, the content of which is presumed to be beer from the German Empire era. Reconstructions of the label refer to the traditional Barre brewery in Lübbecke in northern Germany, which supplied New York and the whole world with beer. A contractually agreed collaboration between the Barre brewery and the Lloyd shipping company in 1885 enabled that every year over 300,000 beers sealed with wax left Germany (Supplementary Figure S1). The label design and elaborate closure indicate that the found beer dates back to that time but never left mainland Germany. The beer, therefore, is referred to as B1885 in the following. In this work, we report on the archeochemical analysis of this unique historic sample contemporary for the time of the industrialization of brewing.

**Figure 1:**
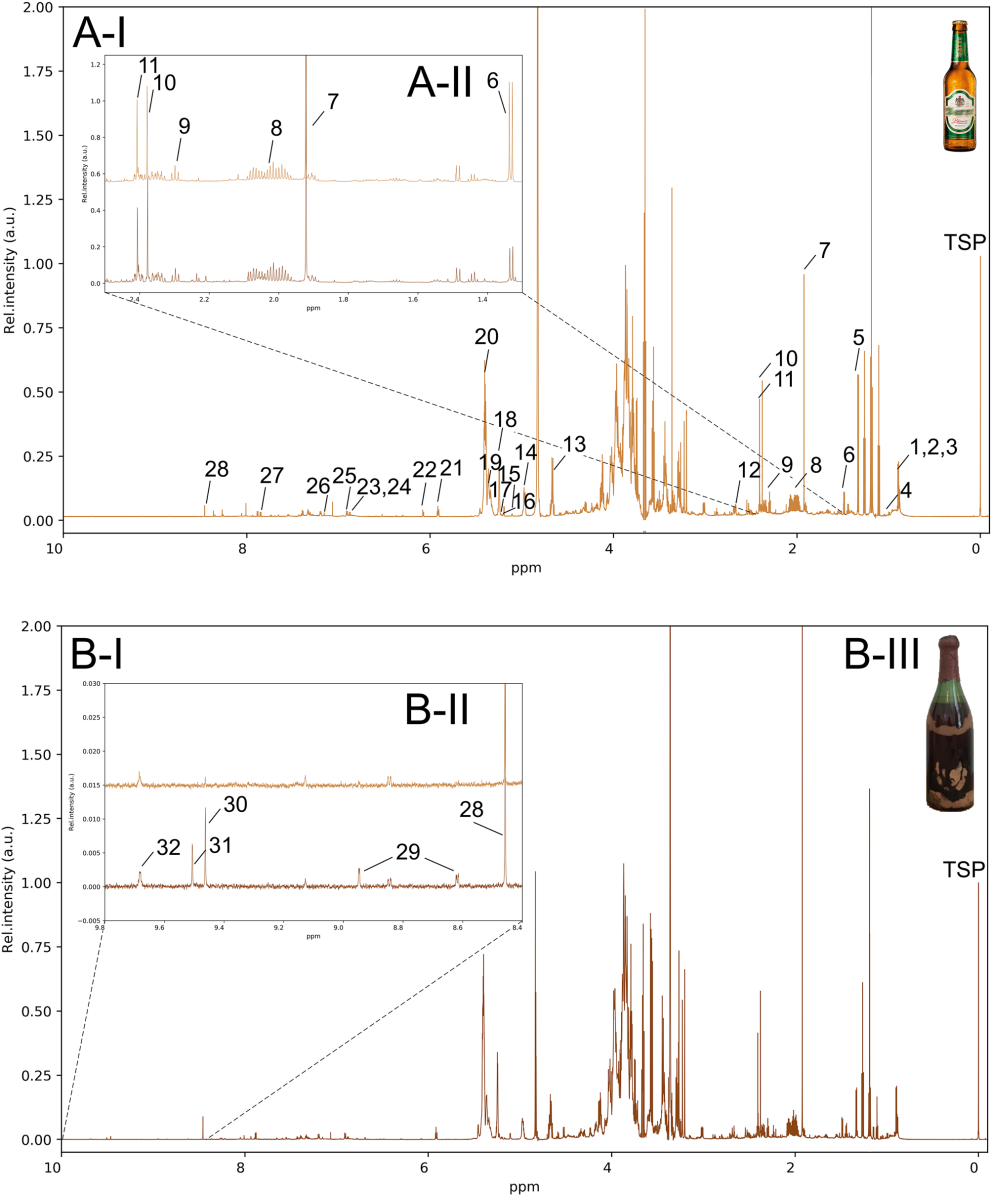
800 MHz 1D-^1^H-NMR spectra of the modern lager beer (**A**-**I**, light brown) and the historical beer (**B**-**I**, dark brown). **A-II** Highlights and compares the regions from 1.3–2.5 ppm containing the signals of small organic acids. **B-II** Highlights and compares the aldehyde region of both beers (modern light brown, top; historical dark brown, bottom). **B-III** The waxed beer bottle from 1885 as it was found. Peak assignments: see Table 1. Peak intensities are normalized to TSP. The pictures of the beer bottles are used under explicit permission of Privatbrauerei Ernst Barre GmbH.

The green bottle (Fig. 1B-III) has a volume of about 0.75 L and was sealed with a cork, fuse wire and wax. The bottle was still a good four-fifths full. The beer had very little sediment. The supernatant was clear and had an amber color. The tasting among four certified tasters resulted in a coherent and well-balanced beer. The smell and taste had sherry and port notes^20^. Likewise, it smelled of prunes. The beer had a slightly weaker palate fullness and still was low sparkling. It was very harmonious in the overall impression and the bitterness.

The classic beer attributes are listed in Supplementary Table S1, compared to other known bottom-fermented beers analyzed in that period and a current Barre beer from 2019 (B2019). The Vienna, Bohemian and Bavarian bottom-fermented beer types popular in the late nineteenth century showed an analytical range of original gravity, alcohol content, real extract and attenuation limit in which the historical beer fit well^21^. The comprehensible and coinciding attributes do not suggest that alcohol has escaped from the bottle in relevant quantities.

With all the characteristics that point to the great preservation of the beer, there were also references to the aging process that the beer has undergone. The color is expected to have been lighter originally. Chemical changes of hop components over the course of about 130 years could lead to altered bitter units. Although it would no longer be appropriate to call the beer a Pilsner from today’s legal point of view, at over 18 IBU, the bitter units still turned out surprisingly high. Yet, not enough to be allowed to call it Pilsner from today's legal point of view. Despite the light-protected surroundings of the finding and the bottling of the beer in a sealed brown glass, oxidation sensitive vitamin B9 folates^22^ were almost entirely degraded when compared to fresh beer samples^23^ (Supplementary Table S1). The nearly optimal conditions, apart from oxygen left in the headspace and the temperature fluctuations, were therefore not sufficient to protect these compounds from aging over many years.

### Microscopy, microbiological cultivation, DNA-screening for wort and beer-related microbes

No yeast and bacteria could be detected via microscopic analysis of the 1885 beer, neither could be cultivated via applied cultivation methods nor could any DNA of specific target DNA-sequences be amplified. Hence, living beer-associated microbes and non-fragmented target DNA could not be detected in the analyzed sample volume. Microscopic analysis revealed no yeast cells-like, rod-like and cocci-like structures and other microbe-like structures. Therefore, we suppose that the 1885 beer was filtered. In unfiltered old beer samples, microbe-like structures like yeast cells are reported to still be visible^14,24,25^. We also suppose that no post-filtration/bottling-derived/cork-derived contamination with beer spoiling microbes took place because no traces for wild yeast, super-attenuating yeast, lactic acid bacteria, acetic acid bacteria, and brewing background bacteria could be detected using the applied methods. Despite analyses of various genomic markers for bottom-fermenting lager yeast *S. pastorianus* and top-fermenting Ale yeast *S. cerevisiae* and a very low detection limit of those qPCR based systems, there was no evidence for brewing yeast in quantities above the detection limit. We suppose a rather efficient sedimentation and filtration process, a few years after the first filtration apparatus was invented by Enzinger^26^. After filtration, during storage, a complete DNA-fragmentation of residual yeast cells took place. Single amorphous cloudy particle structures could be observed via phase-contrast microscopy (magnification 1000-fold) with a size between approx. 5 and 180 µm (Supplementary Figure S2). The structure of the amorphous particles is typical for polyphenol-protein complexes that cause opalescence to turbidity when their concentration increases during beer aging. The amorphous particles were partially dissolvable in 10% KOH and completely dissolvable in concentrated sulphuric acid which indicates the protein fraction of the particles and their organic nature.

### Persistent metabolome and ravages of time revealed by ^1^H-NMR

The 1D-^1^H-NMR spectra of the nineteenth century beer (Fig. 1B-I) and the Barre brewery’s modern equivalent (Fig. 1A-I) are shown in Fig. 1. The overall signature indicates two beer samples that are characterized by a large similarity of metabolite signals, compiled in Supplementary Table S2. The aliphatic region of the spectra (0–3 ppm) showed signals originating from alcohols (ethanol, iso-butanol, iso-pentanol), amino acids (alanine, proline, γ-aminobutyric acid, valine), small organic acids (acetate, lactate, succinate, pyruvate, maleic acid, citric acid) and fatty acids. The midfield region (3–6 ppm) was mostly characterized by carbohydrate signals such as fermentable sugars (glucose, maltose), sugar derivatives (kojibiose) and differently branched dextrins. The aromatic region (6–9 ppm) showed signals of aromatic amino acids (phenylalanine, tryptophan, tyrosine), heterocyclic aromatic compounds (nucleosides, niacin) and polyphenolic compounds that caused the underlying background from which the defined signals rise (6.8–7.5 ppm)^27^. One of the more conspicuous regions was that of aldehydes (> 9 ppm) featuring signals of Maillard- and caramelization-derived 5-hydroxymethyl-2-furaldehyde (HMF) and 4-Hydroxy-2,5-dimethyl-3(2H)-furanon (furaneol). Overall, the qualitative metabolome signature resolved by ^1^H-NMR showed a plurality of matching signals between both samples, underlining their great similarity even after more than 130 years. Differences associated with years of storage and the historical brewing method were, with the exception of few specific signals, primarily determined by the quantitative variance in the signal intensities (Table 1).

**Table 1:** Quantitative determination and change (B1885/B2019) of compounds identified in B1885 and B2019 with ^1^H-Shifts of respective characteristic signals.

Compound (no.)	^1^H-Shift (ppm)^a^	Change^b^	Concentration [mM]^d^		Quanti-fication	References
			B1885	B2019		
Acetic acid (7)	1.92 (s)	+++	3.38	1.38	Integral TSP	^27,97,98^
Furfural (31)	9.50 (s)	+++	0.09	n.d	Integral TSP	Std.^e^
HMF (30)	9.46 (s)	+++	0.10	Trace	Integral TSP	^97^
Niacin (29)	8.9 (dd) 8.6 (dd)	+++	0.05	n.d	Integral TSP	Std.^e^
Unknown N-Heterocycle	8.23 (s)	+++				
Unknown N-Heterocycle	8.21 (s)	+++				
Unknown N-Heterocycle	7.94 (s)	+++				
Acetaldehyde (32)	9.68 (q)	++	0.08	Trace	Integral TSP	^27,97,98^
Formic acid (28)	8.45 (s)	++	0.47	0.21	Integral TSP	^27,97,98^
Kojibiose (15)	5.11 (d)	++				^99^
α-(1–4)-Branched CH^*c*^ (20)	5.35–5.45 (m)	+				^99^
CH^*c*^ reducing end (18)	5.23–5.27 (m)	+				^99^
Glucose (17)	5.19 (d)	+				^99^
Uridine (21)	5.92 (d)	+	0.38	0.30	Line fitting	^27,97,98^
Valine (4)	0.99 (d)	+				^97,98^
2-Methyl-1-propanol (1)	0.88 (d)	0				^27,97,98^
3-Methyl-1-butanol (2)	0.89 (d)	0				^27,97,98^
α(1–6)-branched CH^*c*^ (14)	4.95–5.00 (m)	0				^99^
Alanine (6)	1.48 (d)	0				^27,97,98^
β-branched CH^*c*^ (13)	4.40–4.85 (m)	0				^99^
Citrate (12)	2.53 (d), 2.66 (d)	0				^27,97,98^
GABA (9)	2.30 (t)	0	0.55	0.59	Integral TSP	^27,97,98^
Histidine (26)	7.15 (s)	0	0.10	0.13	Integral TSP	Std.^e^
Malto-oligo-CH^*c*^ (19)	5.25–5.38 (m)	0				^99^
Phenylalanine (25)	7.34 (m) 7.43 (m)	0				^97,98^
Proline (8)	1.95–2.1 (m)	0				^27,97,98^
Propanol (3)	0.89 (t)	0				^27,97,98^
Pyruvate (10)	2.37 (s)	0	0.90	0.75	Line fitting	^27,97,98^
Succinic acid (11)	2.40 (s)	0	0.50	0.50	Line fitting	^27,97,98^
*Tyrosol (23)*	6.87 (m) 7.19 (m)	0				^97,98^
Tyrosine (24)	6.91 (m) 7.20 (m)	0				^27,97,98^
Xylose (16)	5.21 (d)	0				^99^
Adenosin/Inosine (22)	6.07 (d)	−−	Trace	0.16	Line fitting	^27,97,98^
Lactic acid (5)	1.33 (d)	−−	0.65	2.09	Line fitting	^27,97,98^
Polyphenols	6.80–7.45 (m)	−−				^27^
Cytidine (27)	6.07 (d) 7.85 (d)	−−−	Trace	0.23	Integral TSP	^27,97,98^

^a^*s* singlet, *d* doublet, *t* triplet, *q* quartet, *dd* doublet of doublets, *m* multiplet.

^b^+++strong increase in B1885, ++increase, +moderate increase, 0 no change, − moderate decrease, −− decrease, −−−strong decrease.

^c^Carbohydrate.

^d^*n.d*. not detected, *trace* found above the limit of detection, but below the limit of quantification, *empty cells* could not be quantified due to overlapping signals.

^e^Identified through spiking of respective standard.

With a slightly higher valine content in the historical beer, similar profiles of free amino acids were found. The role of nucleosides in beer aging has been described as conspicuous in several studies^28–30^, pointing at 5-methylthioadenosine as a potential compound for oxidative staling. The occurrence of this metabolite from the methionine salvage pathway^31^ could not be reproduced by Yao et al.^32^ in a forced-aging study and was not detected by ^1^H-NMR in this work. A higher uridine concentration was found in historical beer with a lower level of adenosine/inosine. Furthermore, three unidentified signals corresponding to N-heterocycles showed high intensities (7.94 (s), 8.21 (s) and 8.23 (s)).

Another compound featuring an N-heterocycle, niacin, was found in high concentration in the historical beer. Norris^33^ reported the niacin content to be decreasing over the advancing industrialization of the brewing process. While the found content of 6.2 mg/L niacin in the historical beer is plausible for a lager beer of the time (compare approx. 10.3 mg/L in a strong beer of 1872^34^), no niacin signal above the detection limit could be found in modern equivalent (Fig. 1B-II). Niacin is stable throughout the brewing process and storage^35^, directly correlates with the gravity of the beer and is not produced during fermentation in considerable amounts^33,36^. Its low content in nowadays beers cannot be attributed to higher concentrations in historical barley cultivars with levels being consistent between 80 and 120 µg/g^33,34,37,38^. The accumulation of niacin in the germ layers of the barley grain^39^ indicates that the germ was not or insufficiently removed in the historical brewing process.

With a similar overall carbohydrate profile, more α-(1–4)-branchings of dextrins were found, which could be attributed to the differences in the brewing barley or enzyme activities in the historical beer. An increased ratio of reducing α-ends can be attributed to long-term storage, comparable with the finding of oligosaccharide breakdown by Walther et al.^15^. The higher amount of monomeric glucose, as also found for other historical beers^15,16^, could be explained by the same reason or incomplete fermentation with the specific yeast used at the time. The caramelization or Maillard reaction derivatives of such reactive sugars, like kojibiose, consequently showed higher signal intensities after long-term storage.

The observation of decreasing signatures of polyphenols during non-optimal storage of beers has already been described in several studies^27,40^ and was attributed to the reaction of polyphenols with free radicals, reactive oxygen species and acid-catalyzed polymerization^41^. Resulting polymers interact with proteins and form insoluble complexes and hazes, following the non-biotic sediment and microscoped particles settled in the beer bottle. One compound found to promote this process is acetaldehyde. Formed by yeast fermentation or ethanol oxidation, acetaldehyde induces ethyl bridges between the flavanols^42^. Forced aging studies did not show great alterations in the acetaldehyde concentration with a tendency to decrease due to its reactivity^43^. The higher content of this compound in the historical beer, therefore, should be attributed to the control of the fermentation process and the yeast used at the time. Formic acid as another fermentation by-product, as well, is significantly accumulated.

A large increase was found for the acetic acid signal (Fig. 1A-II). At around 155 mg/L, the acetic acid content is slightly above the range that can be expected in today's beer samples^44^ and significantly increased compared to the modern lager with 63 mg/L. Again, the control of the fermentation and the type of yeast used define the acetic acid concentration. Hereby, the amount of yeast, higher fermentation temperature and aeration are beneficial to the acetate content. With a lack of studies on beer, resorting to wine studies^45^, it is reported that the acetic acid concentration remains unchanged during forced aging. The significantly lower lactic acid concentration in the historical beer declines microbial spoilage and thus the origin of acetic acid due to *Acetobacter*.

In nowadays brewing practice, the mash or wort is intendedly acidified by so-called sour wort containing lactic acid to reach pH-values around 5.5 (mash) and 5.2 (wort), respectively. Thereby, optimal enzyme activities, higher degrees of fermentation, protein breakdown, microbiological stability and a lighter color development can be achieved. The low lactic acid concentration indicates that such optimized acidification of the beer has not yet been carried out during historical brewing at the end of the nineteenth century.

The clearest indication of the decades of storage could be found in the area of aldehyde signals (Fig. 1B-II). HMF, generated by multiple pathways during the Maillard reaction and caramelization^46^, was detected in trace amounts in the modern lager beer. The amount of 12.6 mg/L quantified in historical beer exceeds the range of 2 mg/L (pale beer) to 8 mg/L (dark beer) expected for fresh beer of any kind^47^. Numerous forced-aging studies showed that the amount of HMF is independent of the oxygen load and increases significantly with the length of storage^41,48,49^. Another noticeable aldehyde signal could be assigned to furfural. As for HMF, the behavior of the furfural concentration during beer storage was described as increasing at an approximately linear rate with the storage time and exponentially with increasing temperature^49^. Malfliet et al.^50^ reported furfural concentrations between 15 and 35 µg/L in fresh beer. Although force-aged beers, with a maximum observed concentration value around 500 µg/L, never met the taste threshold of furfural (150 mg/L^51^), a clear correlation was found with a staling flavor^52^. The amount of furfural as an indicator compound for beer staling could be quantified to 8.35 mg/L in the historical beer with concentrations below the limit of detection in the modern equivalent. Londesborough et al.^16^ found a level of 664 µg/L furfural in the shipwreck beer from the 1840s that, underwater, was exposed to significantly lower temperatures. The furfural concentration found in the historical beer far exceeds what is described for beer in literature.

### Chemical space of the historical brew resolved by FTICR-MS

The differences in the chemical space and metabolic range between the historical beer and its modern equivalent were investigated by long-time DI-FTICR-MS analysis. The analytical approach offers the unique compositional dimension when chemically characterizing beer samples. After data filtering and annotation through mass difference networks (MDiN), 5200 compositions could be observed for the historical beer (B1885) and 4,250 for the modern equivalent (B2019), respectively. More than 40 molecular formulae could be detected in one nominal mass with great matches between the historical and modern sample (Supplementary Figure S3). The molecular compositions were plotted in van Krevelen diagrams, which have proven to reveal compositional patterns within the metabolite profile of both wine^53–55^ and beer samples^56–58^.

**Figure 2:**
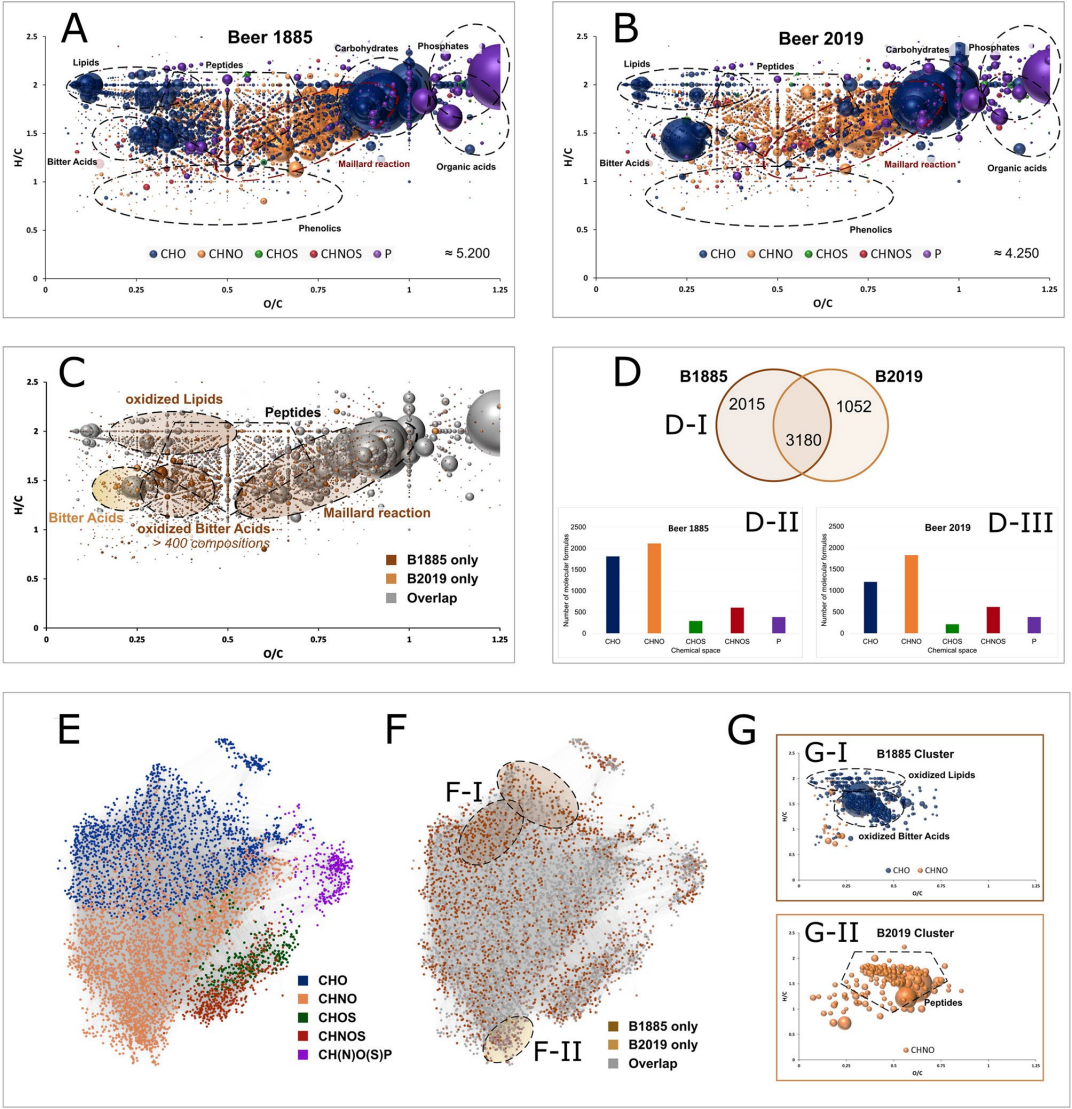
Van Krevelen plots of compositions found in B1885 (**A**), B2019 (**B**), the overlap of the samples (**C**), respective Venn-diagram (**D**-**I**) and chemical spaces for B1885 (**D-II**) and B2019 (**D-III**). Mass difference network of annotated compositions colored by the chemical space (**E**), by presence in sample B1885 and B2019 (**F**) and clusters of compositions specific to B1885 (**F-I**) and B2019 (**F-II**) with their respective position in the van Krevelen diagram (**G-I** and **G-II**, respectively). Color code: CHO (blue), CHNO (orange), CHOS (green), CHNOS (red), CH(N)O(S)P (violet). Neutral compositions are depicted. Approximate regions of compound classes are marked (**A**,**B**,**C**,**G**) and specific areas are highlighted in (**F**).

Comparing the van Krevelen diagrams of both beer samples, it becomes apparent that the compositional space of the 1885s beer (Fig. 2A) shows great overlap with the molecular formulae found in modern beers (Fig. 2B). The dominant carbohydrate cluster (H/C ≈ 2, O/C ≈ 1) is accompanied by respective sugar-phosphates and small organic acids. The degradation of the sugar compounds, associated with the loss of H_2_O, was more pronounced in the historical beer. These degradation processes, usually, are driven by the Maillard reaction and take place during malting and roasting of the grain itself^59^ and are intensified during the brewing process^60^. Taking into account that the beer analyzed was found in the basement of a commercial building, it experienced only moderate temperature fluctuations for around 130 years. The additional sugar-breakdown presumably originates in the chemical changes during the time of storage. At natural room temperature, following unusual reaction conditions for the Maillard reaction in foods and beverages, disproportionately many of the sugar degradation products belonged to the CHO chemical space. In previous studies^58^, analyzing 250 beer samples, one-third of the compositions resulting from the Maillard reaction could be assigned to the CHO- and two-thirds to CHNO-chemical space. In contrast, the chemical spaces are evenly distributed for the sugar degradation compositions only found in the 1885s beer.

Scientific brewing paved its way in those years with the work of Pasteur and Hansen. Without any clear indications for beer pasteurization, Walther et al.^15^ describe great stability of the beer carbohydrates to enzymatic and microbial degradation for beers at that time. The claim for chemical stability is contradicted by the non-enzymatic changes, namely the Maillard compositions described, at least to some extent. The indication of a change in the chemical signature of the beer due to exceedingly long-term storage is mirrored in further compound classes as well. The region of lipids was characterized by more oxygenated species due to oxidation processes (Fig. 2C). Concerning the oxidative alteration of lipids, the formation of (E)-2-nonenal, which is linked to a cardboard-like off-flavor^61^, from linoleic acid is a decisive criterion for the effect of lower oxidation stability in brewing practice^41^. Brewing research largely agrees that the so-called “(E)-2-nonenal potential”^62^ is already generated during the wort production by enzymatic (lipases and lipoxygenases) and non-enzymatic (autoxidation) processes^63^. Further oxidation of the lipids after bottling is considered negligible under normal storage conditions^64,65^. Saturated and comparatively more oxygenated molecular formulae like C_12_H_22_O_5_, C_12_H_24_O_5_ or C_16_H_30_O_8_ as characteristic products in the 1885s beer gave insights into processes that occur during extreme storage times, apart from specific known marker compounds. By hydroxylation (O), chain prolongation (CH_2_), (de)hydrogenation (H_2_) and epoxidation (− H_2_/+O) (bio-)chemical reactions and their combinations, the 150 compositions involved in the oxidation system could be set into relation, leading to a comprehensive mass difference network (Supplementary Figure S4). Glycosylation patterns, of chemical or enzymatic origin, were not found.

The compositions in the area of peptides, more specific to the modern beer, are in agreement with their role in the Maillard reaction. The biggest difference between the samples’ metabolic profiles lay in the region of hops bitter acids. These terpeno-phenolics, which most significantly contribute to the bitterness of beer, showed great presence in both beers, but markedly differed in the degree of oxygenation. The modern and fresh beer spectra, as expected, contained composition signals for the well-known main bitter acids in hops like humulone [C_21_H_30_O_5_], cohumulone [C_20_H_28_O_5_], lupulone [C_26_H_38_O_4_] and colupulone [C_25_H_36_O_4_]. In contrast, there were hundreds of oxygenated derivatives in the historical beer, shifted to the right in the van Krevelen diagram (Fig. 2C). Such an oxidation process could already be indicated^57^, but showed an extraordinary extent in this very special sample. Although the bottle was corked and waxed, which led to a largely maintained ethanol content, the oxygen present in the head-space of the bottle has been sufficient to almost completely oxidize the known hop constituents. Consequently, the signal intensity of [C_21_H_30_O_5_] and [C_20_H_28_O_5_] are drastically decreased. Over 400 new derivative compositions unique for the historical beer were observed. Bearing in mind that several (structural) isomers are to be expected (e.g. at least 12 for humulone itself^66^), the richness of the hops metabolite profile likely even goes far beyond hundreds of compounds. The MDiN between the modern beer hops bitter acids and the derivatives only found in the 1885s beer featured mostly compositional changes equivalent to oxidation reaction (O_3_, O_2_, O_4,_ are the three most common differences), substantiating the assumption of derivative formation through oxidation. These conclusions are well founded by the context of the sample, the positioning of the molecular formulas in the VK diagram, their relationships to each other in the MDiN and the mass difference enrichment analysis. Nevertheless, unambiguous identification (according to Goodacre et al.^67^) and structural information cannot be provided on the basis of accurate mass values. As early as the 1980s, brewing research investigated the degradation of hops on a molecular level to describe the formation of volatile carbonyls, alcohols and esters^68–70^ as ultimate breakdown products. Later, Intelmann et al.^66^ elucidated the molecular structures of several more complex cohumulone derivatives in storage model systems. A quantification method including up to 117 bitter acid derivatives (carboxylic acids, epoxides, cyclic, hydroxylated, and peroxided derivatives) was developed to describe oxidation intermediates and products in hops^71^, throughout the brewing process^72^ and during storage experiments^73^. The beers found in a shipwreck in the Baltic Sea and originating from a similar period were examined using these methods^16^. Comparable to the low signals found in the 1885s beer, negligible amounts of intact α- and β-acids were found. Isomerized humulones were present in minimal amounts. In line with their model experiments, cyclic oxidation products could be identified as a sign of long-term storage. The strong bacterial influence, the impact of low pH and the diffusion of seawater in the Baltic beers surely resulted in special reaction conditions. Nevertheless, it is noticeable that, despite the already comprehensive targeted analytical approach, the hop compounds found in the Baltic beer cover less than 5% of the chemical compositions described in our work. The resolved complexity and richness of hop-derived compounds in the well-preserved historical beer remains a unique description of hops oxidation. It gives important insights into chemical alteration of the hop metabolome over a century in such a 0.75-L micro laboratory that never can be replicated entirely accurately by forced aging experiments.

Overall, compared to the oxidation of wine^54,74^ where sulfur compositions play a major role as antioxidants, the differences between the modern and the 130 years aged historical beer are mostly limited to the CHO-chemical space (Fig. 2D). With more than 60% of the around 5200 molecular formulas overlapping between the found beverage and the modern beer, there should be no doubt that the bottle contains a beer whose age is reflected in oxidation processes. The allocation of the aging products with regard to their chemical origin could also be traced in the MDiN (Fig. 2E–G). It showed distinct areas for the chemical spaces with slight overlapping of the CHO/CHNO and CHOS/CHNOS spheres, respectively (Fig. 2E). The compositions characteristic of the historical or modern beer (Fig. 2F) formed specific clusters in the linked network that corresponded to the described compositional spaces of oxidized lipids, oxidized bitter acids and peptides (Fig. 2G). These findings underline the remarkably good preservation of the beer over 130 years and indicate that, apart from extensive oxidation primarily of the hops components, its metabolic signature is very comparable to modern, industrially brewed beers.

### Chemometric interpretation of the metabolic signature

**Figure 3:**
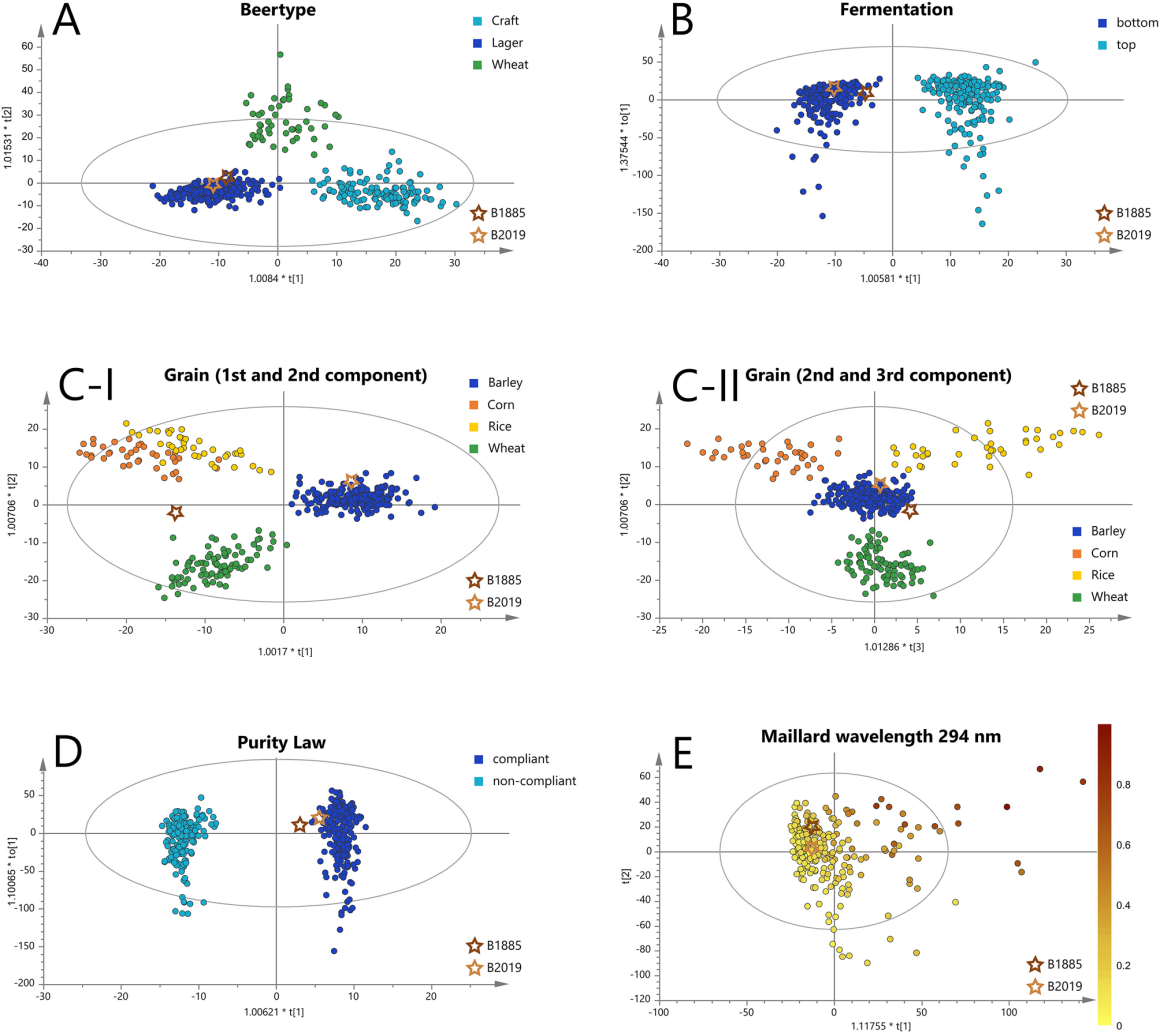
Score plots of the OPLS-(DA) differentiating beer types (**A**), fermentation types (**B**), compliance with the German Purity Law (**C**), grains used (**D**) and Maillard signatures (**E**). The spot for each beer is colored according to its respective class. The position of beers B1885 (dark brown star) and B2019 (light brown star) is based on a prevision based on the statistical model and is indicated by a star.

The beer attributes, the sensory characterization**,** NMR-profile and FTICR-compositions of the historical beer overlap in many parts with today's beer. Given these clear similarities, the metabolic fingerprint of the historical beer was statistically compared with that of hundreds of other modern beers to conclude about its original nature. For this purpose, OPLS-(DA) models based on the DI-FT-ICR-MS (400 scans) molecular profiles of up to 400 beers were developed. The discriminating characteristics (y-variable) were the beer type, the type of fermentation, compliance with the Purity Law, the grain used and the Maillard signature (Fig. 3). All models showed a clear classification power of the samples with regard to the examined criterion. Their statistical relevance concerning the goodness of the fit, quality of prevision and the exclusion of overfitting could be proven with R^2^Y (cum) values between 0.87 and 0.97, Q^2^ (cum) between 0.57 and 0.81 and ANOVA p values ≪ 0.05 respectively^75–77^ (Supplementary Table S3). Based on these models, the most significant compositions could be extracted in the associated loadings plots (Supplementary Figure S5) and visualized in van Krevelen diagrams (Supplementary Figure S6). The underlying chemical information of the statistical models was used to locate the historical beer within the score plots. The position of the equivalent modern beer of the same brewery and known metadata was predicted as well. The great similarity already shown between the well-preserved bottle and industrially manufactured beer was reflected in the fact that the historical sample did not appear as an outlier in any of the models even after ~ 130 years of storage (Hoteling's T^2^). It enabled us to use the molecular fingerprint of the beer to draw conclusions about the brewing method in the nineteenth century when compared to validated metabolic profiles of hundreds of modern beers.

### A typical lager beer

The use of specific yeasts, malts, adjuncts and/or the type of hopping defines the type of beer resulting from the brewing process. These characteristics influence the metabolic signature of the respective way of brewing. As reported earlier^56,57^, wheat beers showed a network of compositions that can be traced back to secondary metabolites (phytoanticipines) of the wheat plant. The major difference between the molecular fingerprint of lager and craft beers is due to the different way of hopping. Dry-hopped craft beers featured a variety of oxidized bitter acid derivatives, whereas the lager and wheat beers showed no defined signature of hops components (Supplementary Figure S6A). Despite the numerous characteristic oxidation products found in both the craft beers and the historical beer, the latter clearly could be assigned to the lager beer type (Fig. 3A). Discrepancy is to be found in the different oxidation mechanisms coming with dry-hopping compared to long-term storage and the associated extraction of hops polyphenols.

Another fundamental difference between the beer types is the type of yeast. Craft beers are fermented with ale yeasts whereas lager beers are brewed with bottom-fermenting yeasts, which were causative for a metabolite pattern of CHNO compositions in the shared region of lipids and amino acids/peptides in the van Krevelen diagram (Supplementary Fig. 5A–I). Only 19 of the respective 112 m*/z*-values showed a database entry (HMDB, YMDB, ChEBi, Metacyc, Lipid maps) with suggested carnitine, ethanolamine and amino acid acyl-conjugates of fatty acids. Despite the yet unknown identity of these compounds, the same signals could be found in both the historical (80%) and modern (87%) beer. The beer of 1885 could be identified as a typical lager beer by the fingerprint of its “dark metabolome”.

### Bottom-fermenting yeast

Although no viable yeast cells could be isolated, it was possible to determine the type of yeast used at the time by its influence on the beer metabolome. In general, when it comes to brewing, a distinction is made between top- and bottom-fermenting yeast species (*Saccharomyces cerevisiae*). They differ in their sprouting and thus the behavior during fermentation^44^. Top-fermenting yeasts in ale or wheat beers form sprouts that rise to the top at the time of the most intensive fermentation. Bottom-fermenting yeasts linger as single cells or cell pairs at the bottom of the fermentation vessel. The biggest differences of brewing-relevance concerning the metabolism are the enzyme expression (e.g. hydrolysis and decarboxylation of ferulic acid to 4-vinylguaiacol [C_9_H_10_O_2_] for wheat beer yeasts^78^) and their optimum temperature. Top-fermented brewing takes place at around 18 °C, whereas the bottom-fermented method prefers cooling to 9 °C. Due to the necessity of elaborate cooling with ice in winter and no such possibility in summer, the bottom-fermented lager spent a little pronounced existence until the second half of the nineteenth century^79^. It was only with the work of Linde, leading to the refrigeration apparatus in the 1870s^10^, that bottom-fermenting yeast was made practicable all year round. It remains unclear whether this groundbreaking invention has already come into use for the historical beer. Yet, the tradition that the associated brewery already had a Linde refrigeration apparatus in 1881^80^ is substantiated by our findings. The metabolic profile could clearly be assigned to that of a bottom-fermented beer (Fig. 3B). The availability of controlled cooling opened up the world of standardized fermentation. The historic brew may be among the first lagers that spread consistent brewing quality and a recognizable taste around the world. It is questionable whether the yeast used was a pure cultured yeast, as the first isolation of single cells was achieved only a few years before by Hansen^9^ during his beer research. Walther et al.^14^ report the genome sequence of the first pure cultured *Saccharomyces carlsbergensis* and report the oldest yet-known beer brewed with this yeast^15^. However, by analyzing similar reference beers (from 1880 to 1990s), the latter authors were able to point out that beer spoilage by wild yeasts was still common in that period.

### Simply Barley

The grain used for brewing serves primarily as a starch and enzyme source and thus as a supply of fermentable carbohydrates. Yet, in addition to these products of primary metabolism, secondary metabolites that are extracted during the brewing process contribute to the molecular diversity of the final beverage. Utilizing the FTICR-MS analytical approach, the molecular profiles of barley, wheat, corn and rice could be characterized and potential marker substances identified using UPLC-ToF–MS^56^. We used these statistical models to examine the metabolic profile of the beers with regard to the use of the various starch sources that still are very common today. The prediction of the modern beer showed a clear allocation to the beers made from pure barley in the score plots of both the 1st against 2nd (Fig. 3C-I) and the 2nd against 3rd (Fig. 3C-II) principal components. In contrast, the historical beer was unambiguously identified as beer without wheat, corn or rice added only in the second score plot. For this reason, subsequent UPLC-ToF-MS measurements were carried out. Neither the characteristic benzoxazinones of wheat (e.g. MBOA, HBOA-Glucoside, DIBOA-glucoside, HMBOA-glucoside), the hydroxyoxindoleacetic acid or the lipid profile of corn nor the rice-specific aspartic acid conjugate of N-glucosyl-indoleacetic acid were found in both beer samples (Supplementary Figure S7). Consequently, using complementary and comprehensive mass spectrometric approaches, it could be demonstrated that the historical beer did not show any metabolites or metabolic signatures that would suggest the use of wheat, corn or rice.

### Brewed according to the Bavarian Purity Law

In the tradition of the Bavarian Purity Law (1516), to this day the use of raw grain, additives and adjuncts, starch and sugar or spices is prohibited in Germany and a few other countries. The chemometric classification of the 400 beers analyzed was based on current law. The beers declared as not compliant with the Purity Law were (1) brewed with corn, rice, soy, raw barley/wheat/rye/oat, malt extracts and syrups, sugar, sugar syrups or starch (2) sweetened with one of the above, caramel or sugar substitutes (3) preserved with antioxidants, stabilizers and acidity regulators (4) made by adding green tea, lotus blossoms, hemp, seaweed, whiskey or brandy (5) refined by yuzu, honey, plumb, cherry, orange peel, chestnut or coffee or (6) flavored with coriander, anise or herbs. The chemical profiles of all these attributes were compared to those beers brewed according to the Purity Law have in common (Fig. 3D). The chemometric analysis of the historical beer’s metabolome suggests it was brewed according to the standards of the Purity Law that is currently in force. Accordingly, in view of the fact that no wheat signature could be identified, it also complied with the regulations of the German imperial era. The production of wheat containing beer was an exclusive right of the Duke and was not allowed to be widely practiced in order to have the wheat reserved for bakers.

### Moderate roasting signature

The last OPLS model was created based on a continuous y-variable. The metadata used was obtained from UV/Vis-measurements like described in an earlier study^58^. The Maillard roasting signature of the historical find was slightly more pronounced, but similar to that of modern pale beers (Fig. 3E). The metabolic signature described a typical pale lager beer, whose Maillard chemical imprint originates not in the roasting process, but long-term storage under moderate conditions.

## Conclusion

**Figure 4:**
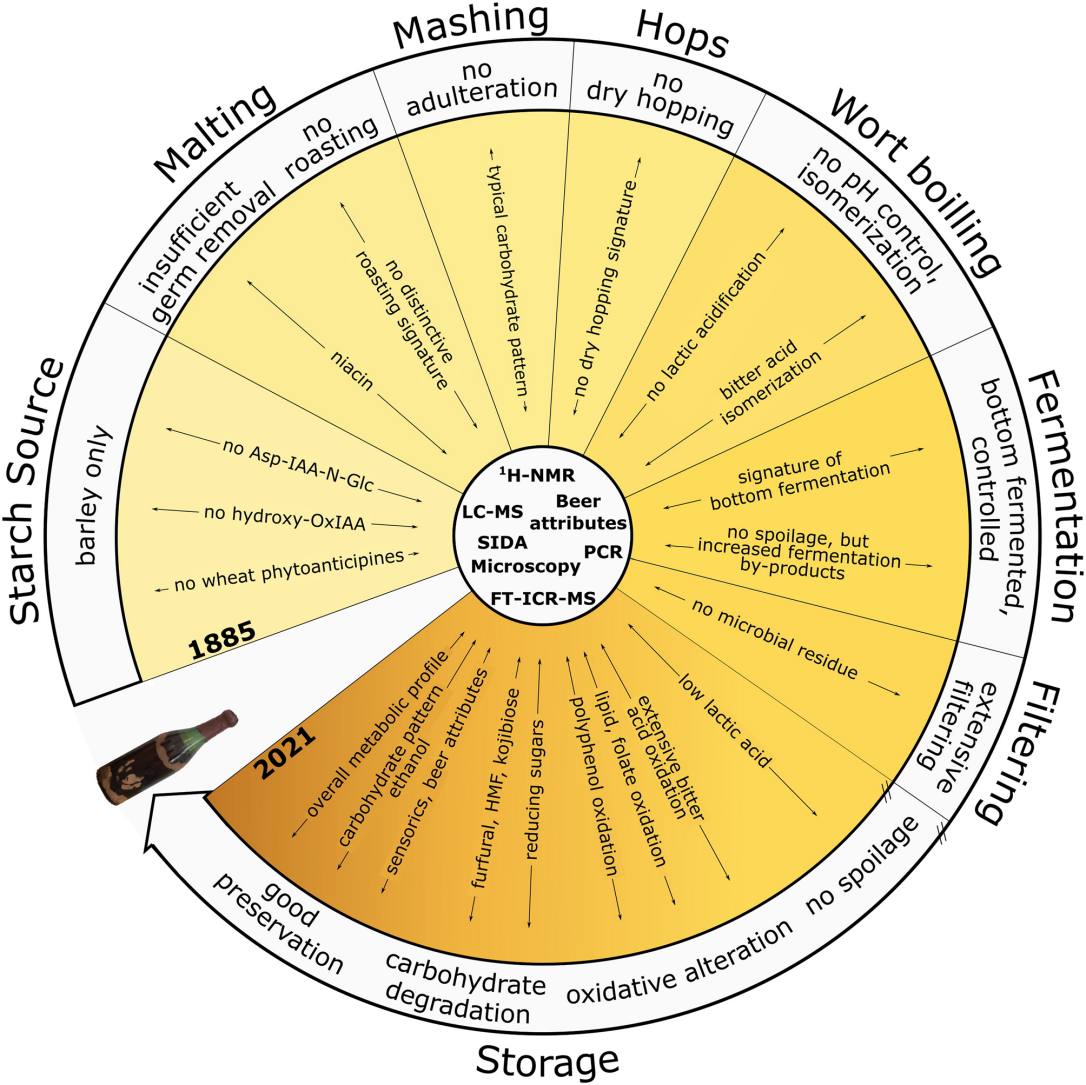
Representation of production steps during the putative brewing process of the historical beer of 1885. The picture of the beer bottle is used under explicit permission of Privatbrauerei Ernst Barre GmbH.

Every raw material involved in the beer-making, the brewing method itself and all production steps towards the type of storage influence the chemical composition of the beer and preserve a specific metabolite footprint. Through comprehensive archeochemical investigations, we showed that the molecular profile of beer can be revealed and interpreted even after more than a 100 years of natural occurring alterations (Fig. 4). The historical brewing process and the changes caused by aging could be described on a molecular level in more detail. We described a hitherto unknown diversity (> 400 specific compositions) of oxidized hops bitter acid derivatives and lipid oxidation (FTICR-MS), the role of niacin as an indicator compound of insufficient germ removal and undescribed high concentrations of Maillard-reaction marker molecules (NMR). The clear indicators of the ravages of time, however, have not been able to obscure the detailed molecular information of the brewing of the late nineteenth century. Despite the over 130 years of storage of the beer under atmospheric pressure and in a standing position, the beer’s original nature was unchanged in many parts. (Ultra)high resolution mass spectrometry enabled the description of the largely unidentified “dark metabolome” of the historical beer and to compare it to modern brewing. In this way, the beer sample could be identified as a typical lager beer, which was subjected to bottom-fermentation even at a time when industrial production with accordant yeasts was still under early development. Following the Bavarian brewing tradition, the Purity Law applicable at the time was complied with, and specific metabolite profiles of adjuncts like wheat, corn or rice could not be detected. Critical points during the historical brewing process could be unraveled by forensic archeochemistry utilizing whole systems’ fingerprints and specific molecular indicators.

## Materials and methods

### Brewing parameters, folate analysis and sensory characterization

Alcohol content and specific gravity were analyzed according to MEBAK (Central European Commission for Brewing Technical Analysis) WBBM (wort, beer, mixed beer beverages) 2.9.6.3 with an Alcolyzer Plus with a DMA 5000 density meter and Xsample 122 sample changer (Anton-Paar GmbH, Ostfildern, Germany) and the pH value according to MEBAK WBBM 2.13. Final attenuation was determined according to MEBAK WBBM 2.8.1. Foam stability was determined according to MEBAK WBBM 2.18.4. Sensory Analysis was performed according to MEBAK II 2.34.3. Samples of the same beer were subjected to forced aging by shaking them overhead for 24 h and storing them at 40 °C for 4 days. The beers were tasted and judged among four certified tasters according to MEBAK II 2.34.3. Folate analysis was performed as described in Pferdmenges et al.^23^ on a Shimadzu Nexera X2 UHPLC system (Shimadzu, Kyoto, Japan), utilizing stable-isotope dilution (Supplementary Table S4).

### Microscopy, microbiological analyses, PCR-based methods

25 mL homogenized sample of the 1885 beer were transferred aseptically to a sterile 50 mL cell culture centrifuge tube. 1 mL of the beer each were transferred to broth-based (liquid) and agar-plate (solid) based cultivation methods. A broad range of culture media for cultivation of beer, wort and beverage related microbes were selected for this approach: Wort-Agar, Wort, YM broth, YM-Agar, YGC-Agar, NBB-Agar, NBB broth, MRS broth, MRS-Agar, Micro Inoculum Broth (MIB), DEV-Nutrient-Agar, DEV-Nutrient broth, PCA, TSA, WLN-Agar, WLD-Agar, YPM broth, OSA, VRBD-Agar, Lactose-Peptone broth. Culture techniques, incubation conditions and incubation periods were applied according to MEBAK III 10.3–10.6, 10.1 and according to Back^81,82^. Additionally, the beer sample was analyzed microscopically according to the method MEBAK III 10.11.3 (using a Microscope Nikon Eclipse E200 with 1000-fold magnification as phase-contrast and dark-field microscopic application). After DNA-extraction of the beer sample-specific Real-Time PCR systems for beer-related yeast and bacteria species (e.g. *Saccharomyces cerevisiae*, *Saccharomyces pastorianus*, other *Saccharomyces* species, non-*Saccharomyces* beer associated yeast species, lactic acid bacteria) and PCR of 16S rDNA (bacteria) and D1/D2 26S rDNA and ITS1-5.8S-ITS2 rDNA (yeast/fungi) with subsequent Sanger-sequencing were carried out according to Brandl^83^, Hutzler^84,85^, Koob et al.^86^, Riedl et al.^87,88^, Sampaio et al.^89^, Schneiderbanger et al.^90^.

### NMR-analysis

The samples of both analyzed beers were diluted 3:1 with D_2_O containing sodium 3-(trimethylsilyl)propionate-d4 (1.8 mM) as a chemical shift reagent and Di-sodium hydrogen phosphate (1.5 M, pH7) to buffer the sample at pH 7.

Experiments were carried out on an 800 MHz Bruker AVANCE lll spectrometer equipped with a 5 mm QCI-probehead at 300 K. 1D ^1^H-spectra were recorded using a 1D version of the nuclear Overhauser effect (NOE) experiment with a shaped pulse for off-resonance presaturation of the ethanol and water signal during the relaxation delay and mixing time. 2D-Experiments consisted of a phase-sensitive TOCSY with shaped off-resonance presaturation and a dipsi mixing scheme^91–93^. HSQC spectra were recorded with a phase-sensitive version using Echo/Antiecho-TPPI gradient selection, decoupling during acquisition and off-resonance presaturation with a shaped pulse during the relaxation delay^94–96^. The assignment of the observed signals was carried out based on of 2D-NMR experiments considering published information^27,97–99^ and spiking of standards, compiled in Supplementary Table S2. Quantification was done by integration of the peaks in the case of isolated peaks and via peak fitting (assuming a Lorentzian peak shape) in the case of overlapping peaks. The obtained areas were used to calculate the corresponding concentration by comparison with the TSP area. Detailed experiment parameters are given in Supplementary Table S5.

### Sample set and FTICR-measurements

A total of 400 samples of commercially available beers from over 50 different countries were analyzed as a basis for statistical modeling. They are predominantly consistent with those chemically characterized in previous works^56–58^. The sample set represents a cross-section of all possible combinations of beer styles, fermentation types, raw materials, color impressions and alcohol contents available to exclude co-varying metadata. The samples were stored, prepared and measured on a Bruker solariX ion cyclotron resonance Fourier transform mass spectrometer (Bruker Daltonics GmbH, Bremen, Germany) as reported recently^56–58^ and summarized in Supplementary Table S4. The obtained raw data was processed as reported^58^ considering the CHNOSPCl-chemical space. Reasonable chemical constraints were set for data filtering (element count: S + P + Cl < 3) resulting in 7,700 unambiguous molecular formulae with a mass error of <  ± 0.15 ppm (at a resolving power of 400,000 at *m/z* 400) as a basis for statistical modeling. An overview of the sample set is given in the Supplementary information (Supplementary Table S6).

The sample set was accompanied by an historical beer from 1885 (B1885) and from the modern lager from 2019 (B2019) to investigate their molecular signature based on single-spectra comparison and statistical prevision of their metabolite profile. The modern beer was kindly provided by the same brewery to which the old beer is assigned and analyzed immediately upon receipt. The beer from 1885 was sampled through a previously disinfected (MeOH, heat) metal syringe. The sampling was carried out through the cork. Care was taken not to transfer any parts of the wax coating. Both these individual samples were measured as referenced above (400 scans for OPLS-(DA)-based metabolic fingerprinting) and additionally with an increased number of 2000 scans for single spectra comparison.

### FTICR data visualization and statistical treatment

For each metadata criterion, the beer type, type of fermentation, compliance with the German Purity Law, grains used and wavelength at 294 nm (Maillard signature), we performed a supervised OPLS analysis on the FTICR dataset. Based on these models, the position of beers B1885 and B2019 in the score plots were determined. A sevenfold cross validation (R^2^Y cum, Q^2^ cum) and additional CV-ANOVA were performed to validate the models’ significance. The statistical parameters of the beer samples (Supplementary Table S7) and OPLS models (Supplementary Table S3) can be found in the Supplementary information.

The characteristic composition profile for each observation and the compositions found in the 2000 scan spectra of B1885 and B2019 were plotted in van Krevelen diagrams. By plotting H/C versus O/C atomic ratios it is possible to depict common compositional patterns within observations’ markers^53,100,101^. It enables tentative classification of the metabolite signals resolved^101^. The specifically addressed areas of hop bitter acids and lipids^101^ were validated by plotting the respective HMDB^102^ entries. The region of the Maillard reaction is based previous model studies^103^. The peptides compositional area was validated by plotting all theoretical peptides within the 1.000 Da mass range. Compositions characteristic for certain beer attributes were subjected to database search including HMDB^102^, YMDB^104^, ChEBi^105^, Metacyc^106^, and Lipid maps^107^. A mass difference network (MDiN) was applied utilizing the NetCalc approach^108^. The nodes, representing the annotated sum formulae, were connected by edges that represent compositional changes corresponding to 250 different (bio)chemical reactions.

### UHPLC-ToF–MS measurements and marker compound comparison

As described earlier^56^, the statistical analysis of a sub-sample set (102 beers) revealed compounds characteristic for the use of wheat, corn and rice with identification levels reaching from 1 to 3^109^. Utilizing the same sample preparation and Shimadzu LCMS-9030 Q ToF (Shimadzu Deutschland GmbH, Duisburg, Germany) analytical system, beers B1885 and B2019 were screened for those marker molecules to verify the carbohydrate source used. For comparison, class-QC samples were analyzed containing all wheat, corn or rice samples, respectively. The measurement parameters are summarized in Supplementary Table S4.

## Supplementary Information

Supplementary Information.

## Data Availability

The extensive metabolomic and further raw data supporting the conclusions of this article will be made available by the authors on reasonable request, without undue reservation.

## References

[1] McGovern PE (2009). Uncorking the Past: The Quest for Wine, Beer, and Other Alcoholic Beverages.

[2] McGovern PE (2018). Ancient Brews. Rediscovered and Re-created. Reprint edn.

[3] Dietrich O, Heun M, Notroff J, Schmidt K, Zarnkow M (2012). The role of cult and feasting in the emergence of Neolithic communities New evidence from Göbekli Tepe, south-eastern Turkey. Antiquity.

[4] Shalaby, N. *et al*. The lost papers: Rewriting the narrative of early egyptology with the Abydos Temple Paper Archive. https://www.arce.org/abydos-paper-archive (2018).

[5] Michel RH, McGovern PE (1993). The first wine & beer. Chemical detection of ancient fermented beverages. Anal. Chem..

[6] Duke Wilhelm, I. D. L., X. Bayerische Landesverordnung. Chapter 2, Row 13–17 (1516).

[7] Pasteur L (1861). Comp. Rendus Séances Acad. Sci..

[8] Pasteur, L. in Œuvres de Pasteur (ed V.-R. Pasteur) Ch. Études sur le vinaigre et sur le vin, 352 (Masson, 1924).

[9] Hansen EC (1883). Recherches sur la physiologie et la morphologie des ferments alcooliques. V. Methodes pour obtenir des cultures pures de Saccharomyces et de microorganismes analogous. Compt. Rend. Trav. Lab. Carlsberg.

[10] Linde, C. P. G. Refrigerating and ice making apparatus. US patent US 10522 (1884).

[11] McGovern PE, Hartung U, Badler VR, Sglusker DL, Exner LJ (1997). The beginnings of winemaking and viniculture in the ancient Near East and Egypt. Expedition.

[12] McGovern PE (2013). Beginning of viniculture in France. PNAS.

[13] Ault RG (1965). Spoilage bacteria in brewing—a review. J. Inst. Brew..

[14] Walther A, Hasselbart A, Wendland J (2014). Genome sequence of *Saccharomyces carlsbergensis*, the World’s First Pure Culture Lager Yeast. G3 (Bethesda).

[15] Walther A, Ravasio D, Qin F, Wendland J, Meier S (2015). Development of brewing science in (and since) the late 19th century: Molecular profiles of 110–130 year old beers. Food Chem..

[16] Londesborough J (2015). Analysis of beer from an 1840s' shipwreck. J. Agric. Food Chem..

[17] Pryde J (2011). Sensory and chemical analysis of ‘Shackleton's’ Mackinlay scotch whisky. J. Inst. Brew..

[18] Jeandet P (2015). Chemical messages in 170-year-old champagne bottles from the Baltic Sea: Revealing tastes from the past. PNAS.

[19] Roullier-Gall C, Heinzmann SS, Garcia J-P, Schmitt-Kopplin P, Gougeon RD (2017). Chemical messages from an ancient buried bottle: Metabolomics for wine archeochemistry. NPJ Sci. Food.

[20] Scholtes C, Nizet S, Collin S (2015). How sotolon can impart a Madeira off-flavor to aged beers. J. Agric. Food Chem..

[21] Thausing, J. E. Die Theorie und Praxis der Malzbereitung und Bierfabrikation. 946 (Gebhardt's, 1888).

[22] Delchier N (2014). Thermal degradation of folates under varying oxygen conditions. Food Chem..

[23] Pferdmenges, L. E. *et al.* Characterization of the nutrient composition of German beer styles for the German nutrient database. *J. Food Compos. Anal*. (2021, accepted manuscript).

[24] Thomas K, Ironside K, Clark L, Bingle L (2021). Preliminary microbiological and chemical analysis of two historical stock ales from Victorian and Edwardian brewing. J. Inst. Brew..

[25] Hutzler, M. Yeast biodiversity of traditional and modern hop beer fermentations and their targeted expansion via developed yeast hunting Methods Habilitation Thesis thesis, Technical University Berlin, (2021).

[26] Enzinger, L. E. Apparat mit Filterböden aus Papier zum Filtrieren von trüben Flüssigkeiten. Germany patent (1879).

[27] Duarte I (2002). High-resolution nuclear magnetic resonance spectroscopy and multivariate analysis for the characterization of beer. J. Agric. Food Chem..

[28] Heuberger AL (2016). Evaluation of non-volatile metabolites in beer stored at high temperature and utility as an accelerated method to predict flavour stability. Food Chem..

[29] Heuberger AL (2012). Metabolomic profiling of beer reveals effect of temperature on non-volatile small molecules during short-term storage. Food Chem..

[30] Hughey CA, McMinn CM, Phung J (2016). Beeromics: From quality control to identification of differentially expressed compounds in beer. Metabolomics.

[31] Avila MA, Garcia-Trevijano ER, Lu SC, Corrales FJ, Mato JM (2004). Methylthioadenosine. Int. J. Biochem. Cell Biol..

[32] Yao J, Zong X, Cui C, Mu L, Zhao H (2018). Metabonomics analysis of nonvolatile small molecules of beers during forced ageing. J. Food Sci. Tech..

[33] Norris FW (1945). Nicotinic acid in brewing materials. J. Inst. Brew..

[34] Barton-Wright EC (1944). The microbiological assay of nicotinic acid in cereals and other products. Biochem. J..

[35] Younger M, Harvey EH (1945). Stability of added vitamins in beer. Food Res..

[36] Stringer WJ (1946). Vitamins in beer. J. Inst. Brew..

[37] Ward CM, Trennerry VC (1997). The determination of niacin in cereals, meat and selected foods by capillary electrophoresis and high performance liquid chromatography. Food Chem..

[38] Davis CF, Laotee S, Sealetan L (1943). A study of some of the vitamin B complex factors in malted and unmalted barley and wheat of the 1941 crop. Cereal Chem..

[39] Ball GFM (2005). Vitamins in Foods.

[40] Qureshi AA, Burger WC, Prentice N (1978). Polyphenols and pyrazines in beer during aging. J. Am. Soc. Brew. Chem..

[41] Vanderhaegen B, Neven H, Verachtert H, Derdelinckx G (2006). The chemistry of beer aging—a critical review. Food Chem..

[42] Saucier C, Bourgeois G, Vitry C, Roux D, Glories Y (1997). Characterization of (+)-catechin-acetaldehyde polymers: A model for colloidal state of wine polyphenols. J. Agric. Food Chem..

[43] Vanderhaegen B, Delvaux F, Daenen L, Verachtert H, Delvaux FR (2007). Aging characteristics of different beer types. Food Chem..

[44] Narziss L, Back W, Gastl M, Zarnkow M (2017). Abriss der Bierbrauerei.

[45] Simpson RF (1978). Aroma and compositional changes in wine with oxidation, storage and ageing. Vitis.

[46] Capuano E, Fogliano V (2011). Acrylamide and 5-hydroxymethylfurfural (HMF): A review on metabolism, toxicity, occurrence in food and mitigation strategies. LWT-Food Sci. Technol..

[47] Viegas O, Prucha M, Gökmen V, Ferreira IM (2018). Parameters affecting 5-hydroxymethylfurfural exposure from beer. Food Addit. Contam..

[48] Rakete S, Klaus A, Glomb MA (2014). Investigations on the Maillard reaction of dextrins during aging of pilsner type beer. J. Agric. Food Chem..

[49] Madigan D, Perez A, Clements M (2018). Furanic aldehyde analysis by HPLC as a method to determine heat-induced flavor damage to beer. J. Am. Soc. Brew. Chem..

[50] Malfliet S (2008). Flavour instability of pale lager beers: Determination of analytical markers in relation to sensory ageing. J. Inst. Brew..

[51] Meilgaard MC (1975). Flavor chemistry of beer. II. Flavour and threshold of 239 aroma volatiles. Tech. Q. Master Brew. Assoc. Am..

[52] Li M, Yang Z, Yang M, Shan L, Dong J (2009). Determination of furfural in beer by high-performance liquid chromatography with solid-phase extraction. J. Inst. Brew..

[53] Gougeon RD (2009). The chemical diversity of wines can reveal a metabologeography expression of cooperage oak wood. PNAS.

[54] Karbowiak T (2019). Wine aging: A bottleneck story. NPJ Sci. Food.

[55] Roullier-Gall C, Boutegrabet L, Gougeon RD, Schmitt-Kopplin P (2014). A grape wine chemodiversity comparison of different appelations in burgundy: Vintage vs terroir effects. Food Chem..

[56] Pieczonka SA, Paravicini S, Rychlik M, Schmitt-Kopplin P (2021). On the trail of the German Purity Law: Distinguishing the metabolic signatures of wheat, corn and rice in beer. Front. Chem..

[57] Pieczonka SA, Lucio M, Rychlik M, Schmitt-Kopplin P (2020). Decomposing the molecular complexity of brewing. NPJ Sci. Food.

[58] Pieczonka SA (2021). Hidden in its color: A molecular-level analysis of the beer's Maillard reaction network. Food Chem..

[59] Hellwig M, Henle T (2020). Maillard reaction products in different types of brewing malt. J. Agric. Food Chem..

[60] Nobis A, Wendl S, Becker T, Gastl M (2021). Formation and degradation of 3-deoxyglucosone as a key intermediate for ageing indicators during wort boiling. J. Inst. Brew..

[61] Jamieson AM, Van Gheluwe JEA (1970). Identification of a compound responsible for cardboard flavor in beer. Proc. Am. Soc. Brew. Chem..

[62] Drost BW, van den Berg R, Freijee FJM, van der Velde EG, Hollemans M (1990). Flavor stability. J. Am. Soc. Brew. Chem..

[63] Liégeois C, Meurens N, Badot C, Collin S (2002). Release of deuterated (E)-2-nonenal during beer aging from labeled precursors synthesized before boiling. J. Agric. Food Chem..

[64] Lermusieau G, Noël S, Liégeois C, Collin S (1999). Nonoxidative mechanism for development of trans-2-nonenal in beer. J. Am. Soc. Brew. Chem..

[65] Noël S (1999). The use of Oxygen 18 in appraising the impact of oxidation process during beer storage. J. Inst. Brew..

[66] Intelmann D (2009). Structures of storage-induced transformation products of the beer's bitter principles, revealed by sophisticated NMR spectroscopic and LC-MS techniques. Chem. Eur. J..

[67] Goodacre R (2007). Proposed minimum reporting standards for data analysis in metabolomics. Metabolomics.

[68] Hashimoto N, Eshima T (1979). Oxidative degradation of isohumulones in relation to flavour stability of beer. J. Inst. Brew..

[69] Williams RS, Wagner HP (1979). Contribution of hop bitter substances to beer staling mechanisms. J. Am. Soc. Brew. Chem..

[70] Hashimoto N, Kuroiwa Y (1975). Proposed pathways for the formation of volatile aldehydes during storage of bottled beer. Proc. Am. Soc. Brew. Chem..

[71] Dresel M, Vogt C, Dunkel A, Hofmann T (2016). The bitter chemodiversity of hops (*Humulus lupulus* L.). J. Agric. Food Chem..

[72] Haseleu G (2010). Quantitative sensomics profiling of hop-derived bitter compounds throughout a full-scale beer manufacturing process. J. Agric. Food Chem..

[73] Intelmann D (2011). Comprehensive sensomics analysis of hop-derived bitter compounds during storage of beer. J. Agric. Food Chem..

[74] Nikolantonaki M (2018). Impact of glutathione on wines oxidative stability: A combined sensory and metabolomic study. Front. Chem..

[75] Westerhuis JA (2008). Assessment of PLSDA cross validation. Metabolomics.

[76] Golbraikh A, Tropsha A (2002). Beware of q2!. J. Mol. Graph. Model..

[77] Eriksson L, Trygg J, Wold S (2008). CV-ANOVA for significance testing of PLS and OPLS models. J. Chemom..

[78] Coghe S, Benoot K, Delvaux F, Vanderhaegen B, Delvaux FR (2004). Ferulic acid release and 4-vinylguaiacol formation during brewing and fermentation: Indications for feruloyl esterase activity in *Saccharomyces cerevisiae*. J. Agric. Food Chem..

[79] Meußdoerffer FG (2009). A Comprehensive History of Beer Brewing.

[80] Mattner, W. Ch. 4, 10–11 (Ernst Barre Privatbrauerei, 1992).

[81] Back W (1994). Farbatlas und Handbuch der Getrankemikrobiologie.

[82] Back W (2006). Colour Atlas and Handbook of Beverage Biology.

[83] Brandl, A. Entwicklung und Optimierung von PCR-Methoden zur Detektion und Identifizierung von brauereirelevanten Mikroorganismen zur Routine-Anwendung in Brauereien PhD thesis, Technical University of Munich (2006).

[84] Hutzler, M. Entwicklung und Optimierung von Methoden zur Identifizierung und Differenzierung von getränkerelevanten Hefen PhD thesis, Technical University of Munich (2009).

[85] Hutzler M (2010). Getränkerelevante Hefen—Identifizierung und Differenzierung.

[86] Koob J, Jacob F, Wenning M, Hutzler M (2017). *Lactobacillus cerevisiae* sp. nov., isolated from a spoiled brewery sample. Int. J. Syst. Evol..

[87] Riedl R, Fütterer J, Goderbauer P, Jacob F, Hutzler M (2019). Combined yeast biofilm screening—characterization and validation of yeast related biofilms in a brewing environment with combined cultivation and specific real-time PCR screening of selected indicator species. J. Am. Soc. Brew. Chem..

[88] Riedl R, Goderbauer P, Brandl A, Jacob F, Hutzler M (2017). Bavarian wheat beer process, a special microbe habitat—cultivation, detection, biofilm formation characterization of selected lactic acid bacteria spoilers and hygiene indicators. BrewingScience.

[89] Sampaio JP, Pontes A, Libkind D, Hutzler M (2017). in Brewing Microbiology: Current Research, Omics and Microbial Ecology.

[90] Schneiderbanger J, Jacob F, Hutzler M (2019). Genotypic and phenotypic diversity of *Lactobacillus rossiae* isolated from beer. J. Appl. Microbiol..

[91] Cavanagh J, Rance M (1990). Sensitivity improvement in isotropic mixing (TOCSY) experiments. J. Magn. Reson..

[92] Sklenar V, Piotto M, Leppik R, Saudek V (1993). Gradient-tailored water suppression for 1H–15N HSQC experiments optimized to retain full sensitivity. J. Magn. Reson..

[93] Piotto M, Saudek V, Sklenar V (1992). Gradient-tailored excitation for single-quantum NMR spectroscopy of aqueous solutions. J. Biomol. NMR.

[94] Palmer AG, Cavanagh J, Wright PE, Rance M (1991). Sensitivity improvement in proton-detected two-dimensional heteronuclear correlation NMR spectroscopy. J. Magn. Reson..

[95] Kay LE, Keifer P, Saarinen T (1992). Pure absorption gradient enhanced heteronuclear single quantum correlation spectroscopy with improved sensitivity. J. Am. Chem. Soc..

[96] Schleucher J (1994). A general enhancement scheme in heteronuclear multidimensional NMR employing pulsed field gradients. J. Biomol. NMR.

[97] Rodrigues JA, Barros AS, Carvalho B, Brandão T, Gil AM (2011). Probing beer aging chemistry by nuclear magnetic resonance and multivariate analysis. Anal. Chim. Acta.

[98] Rodrigues JE, Gil AM (2011). NMR methods for beer characterization and quality control. Magn. Reson. Chem..

[99] Peterson BO, Nilsson MB, Hindsgaul O, Meier S (2014). 1H NMR spectroscopy for profiling complex carbohydrate mixtures in non-fractionated beer. Food Chem..

[100] Hertkorn N (2008). Natural organic matter and the event horizon of mass spectrometry. Anal. Chem..

[101] Schmitt-Kopplin P (2019). Systems chemical analytics: Introduction to the challenges of chemical complexity analysis. Faraday Discuss..

[102] Wishart DS, Feunang YD, Marcu A, Gua AC, Liang K (2018). HMDB 4.0—The human metabolome database for 2018. Nucleic Acids Res..

[103] Hemmler D (2017). Evolution of complex Maillard chemical reactions, resolved in time. Sci. Rep..

[104] Ramirez-Guana M (2017). YMDB 2.0: A significantly expanded version of the yeast metabolome database. Nucleic Acids Res..

[105] Hastings J (2013). The ChEBI reference database and ontology for biogically relevant chemistry: Enhancements for 2013. Nucleic Acids Res..

[106] Caspi R (2018). The MetaCyc database of metabolic pathways and enzymes. Nucleic Acids Res..

[107] Sud M (2006). LMSD: LIPID MAPS structure database. Nucleic Acids Res..

[108] Tziotis D, Hertkorn N, Schmitt-Kopplin P (2011). Kendrick-analogous network visualization of ion cyclotron resonance Fourier transform mass spectra: Improved options for the assignment of elemental compositions and the classification of organic molecular complexity. Eur. J. Mass Spectrom..

[109] Sumner LW (2007). Proposed minimum reporting standards for chemical analysis. Metabolomics.

