# Author Correction: Archeochemistry reveals the first steps into modern industrial brewing

**DOI:** 10.1038/s41598-022-19092-w

**Published:** 2022-08-31

**Authors:** Stefan A. Pieczonka, Martin Zarnkow, Philippe Diederich, Mathias Hutzler, Nadine Weber, Fritz Jacob, Michael Rychlik, Philippe Schmitt-Kopplin

**Affiliations:** 1 Chair of Analytical Food Chemistry, Technical University of Munich, Maximus-von-Imhof-Forum 2, 85354 Freising, Germany; 2 Research Unit Analytical BioGeoChemistry, Helmholtz Munich, Ingolstädter Landstraße 1, 85764 Neuherberg, Germany; 3 Research Center Weihenstephan for Brewing and Food Quality, Technical University of Munich, Alte Akademie 3, 85354 Freising, Germany

Correction to: *Scientific Reports* https://doi.org/10.1038/s41598-022-12943-6, published online 03 June 2022

The original version of this Article contained errors in the niacin content units. In the Results and discussion section, under the sub-heading ‘Persistent metabolome and ravages of time revealed by ^1^H‑NMR.’

 “While the found content of 6.2 g/L niacin in the historical beer is plausible for a lager beer of the time (compare 10.3 g/L in a strong beer of 1872^34^), no niacin signal above the detection limit could be found in modern equivalent (Fig. 1B-II). Niacin is stable throughout the brewing process and storage^35^, directly correlates with the gravity of the beer and is not produced during fermentation in considerable amounts^33,36^. Its low content in nowadays beers (5 mg/L^37^) cannot be attributed to higher concentrations in historical barley cultivars with levels being consistent between 80 and 120 µg/g^33,34,38,39^.”

now reads:

“While the found content of 6.2 mg/L niacin in the historical beer is plausible for a lager beer of the time (compare approx. 10.3 mg/L in a strong beer of 1872^34^), no niacin signal above the detection limit could be found in modern equivalent (Fig. 1B-II). Niacin is stable throughout the brewing process and storage^35^, directly correlates with the gravity of the beer and is not produced during fermentation in considerable amounts^33,36^. Its low content in nowadays beers cannot be attributed to higher concentrations in historical barley cultivars with levels being consistent between 80 and 120 μg/g^33,34,37,38^.”

As a result, Reference 37 has been removed and the following references have been re-numbered. The original reference 37 appears below.

37. Punčochářová, L., Pořízka, J., Diviš, P. & Štursa, V. Study of the influence of brewing water on selected analytes in beer. *Potr. S. J. F. Sci.* **13**, 1–8 (2019).

The original Article has been corrected.

