# Supplementary material for: Archeochemistry reveals the first steps into modern industrial brewing
Source: Sci Rep. 2022 Jun 3;12:9251. doi: 10.1038/s41598-022-12943-6 (PMC9166709; doi:10.1038/s41598-022-12943-6)
Supplement: Supplementary file 1 — Supplementary Information. [file 41598_2022_12943_MOESM1_ESM.pdf]

Supplementary Information for

Archeochemistry reveals the first steps into modern industrial brewing

Stefan A. Pieczonka [a,b], Martin Zarnkow [c], Philippe Diederich [b], Mathias Hutzler [c], Nadine Weber [a], Fritz Jacob [c], Michael Rychlik [a], Philippe Schmitt-Kopplin [a,b]

Corresponding Authors: Stefan A. Pieczonka, Philippe Schmitt-Kopplin
Email: stefan.pieczonka@tum.de, schmitt-kopplin@tum.de

This PDF file includes:

Tables S1 to S7
Figures S1 to S7
SI References are included in the captions and headings

Supplementary tables

Supplementary Table S1. Regular beer attributes and folate contents of Barre Pilsener from 1885 and 2019 compared to Vienna, Böhmisch and Bayerisch beer from 1888.

Beer attribute	Unit	Barre Pilsner 1885	Vienna Beer [a]	Bohemian Beer [a]	Bavarian Beer [a]	Barre Pilsner 2019
Original gravity	w/w%	10.36	10.39-13.26	12.45	14.71	11.195
Alcohol	w/w%	3.12	2.9-3.7	3.43	3.94	3.94
Real extract	w/w%	4.26	4.4-5.7	5.4	6.7	3.6
Attenuation limit, real	%	58.8	57	56.2	54.3	68
pH	-	4.42	-	-	-	4.21
Bitter units	IBU [b]	18.4	-	-	-	28.7
Color	EBC [c]	12.5	-	-	-	7.7
**Folate analysis**						
PteGlu	[µg/100g]	n.d.	-	-	-	n.d. [d]
H_4Holate	[µg/100g]	n.d.	-	-	-	n.d.
5-CH_3-H_4Folate	[µg/100g]	0.58	-	-	-	1.10
5-CHO-H_4Folate	[µg/100g]	n.d.	-	-	-	2.06
10-CHO-PteGlu	[µg/100g]	n.d.	-	-	-	3.91
Total Folates	[µg/100g]	0.58	-	-	-	7.08

[a] Thausing, J.E. (1888), Die Theorie und Praxis der Malzbereitung und Bierfabrikation, Gebhardt's

[b] International Bitter Units

[c] European Brewery Convention (value)

[d] not detected

Supplementary Table S2. ^{1}H and ^{13}C chemical Shifts, Proton Multiplicities for identified metabolites in the beer samples***.***

**Compound (No.)**	**Assigned group**	**δ ^{1}H**	**Multiplicity** [a]	**δ ^{13}C**
2-Methyl-1-propanol (1)	2xC**H**$_3$	0.88	d	21.0
	(CH$_3$)$_2$C**H**CH$_2$OH	1.76	m	27.0
	(CH$_3$)$_2$CHC**H**$_2$OH	3.38		
3-Methyl-1-butanol (2)	2xC**H**$_3$	0.89	d	24.8
	(CH$_3$)$_2$C**H**(CH$_2$)$_2$OH	1.44	m	43.0
	(CH$_3$)$_2$CHC**H**$_2$CH$_2$OH	1.66<br>3.64	m	27.2
Propanol (3)	C**H**$_3$CH$_2$CH$_2$OH	0.89	t	n.d. [b]
	CH$_3$C**H**$_2$CH$_2$OH	1.56	m	
	CH$_3$CH$_2$C**H**$_2$OH	3.57	t	
Valine (4)	2xC**H**$_3$	1.05, 0.99 2.27	d, d	19.7
	(CH$_3$)$_2$C**H**		m	
Lactic acid (5)	C**H**$_3$(CHOH)COOH	1.33	d	19.33
	CH$_3$(C**H**OH)COOH	4.11	q	71.6
Alanine (6)	C**H**$_3$	1.48	d	19.4
	C**H**	3.79	q	
Acetic acid (7)	C**H**$_3$	1.92	s	26.3
Proline (8)	γ-C**H**$_2$	2.00	m	26.6
	β-C**H**$_2$	2.06	m	31.7
	β-C**H**$_2$	2.33	m	31.7
	σ-C**H**$_2$	3.32	m	48.9
	σ-C**H**$_2$	3.41	m	48.9
	α-C**H**	4.11	dd	64.1
GABA (9)	C**H**$_2$COO	2.30	t	37.3
	(CH$_2$)C**H**$_2$(CH$_2$	1.90	m	26.7
	NH$_2$C**H**$_2$	3.02	t	42.3
Pyruvate (10)	C**H**$_3$	2.37	s	29.5
Succinic acid (11)	(C**H**$_2$)$_2$	2.40	s	37.4
Citrate (12)	C**H**´$_2$	2.53	d	48.8
	C**H**´´$_2$	2.66	d	48.8
(13) – (20)	Compound classes			
Uridine (21)	2x C=C**H**	5.92, 7.88	d, d	90.7,144.8
Adenosin/ Inosine (22)	N=C**H**´	8.27	s	155.7
	N=C**H**´´	8.36	s	143.5
	OC**H**N	6.07	d	91.3
Tyrosol (23)	2x C=C**H**´	6.87	m	118.6
	2x C=C**H**´´	7.19	m	133.4
Tyrosine (24)	2x C=C**H**´	6.91	m	119.0
	2x C=C**H**´´	7.20	m	133.7
Phenylalanine (25)	C**H**´$_{aromatic}$	7.34	m	132.1
	C**H**´´$_{aromatic}$	7.43	m	131.1
Histidine (26)	N=C**H**	8.00	s	140.7
	C=C**H**	7.06	s	119.5
Cytidine (27)	C=C**H**´	6.07	d	99.2
	C=C**H**´´	7.85	d	144.7
Formic acid (28)	**H**COOH	8.46	s	174.0
Niacin (29)	4x C=C**H**	8.95, 8.62 8.27,	m	n.d.

Supplementary Table S2 (continued). ^{1}H and ^{13}C chemical Shifts, Proton Multiplicities for identified metabolites in the beer samples.

Compound (No.)	Assigned group	σ ^{1}H	Multiplicity [a]	σ ^{13}C
HMF (30)	C**H**O	9.46	s	183.7
	2x C=C**H**	7.55	d	n.d.
	C**H**$_2$OH	6.69	m	n.d.
Furfural (31)	C**H**O	9.50	s	n.d.
	3x C=C**H**	7.94, 7.60, 6.78	m	n.d.
Acetaldehyde (32)	C**H**O	9.68	q	n.d.
	C**H**$_3$	2.21	d	n.d.

[a] singlet (s), doublet (d), triplet (t), quartet (q), doublet of doublets (dd), multiplet (m)

[b] not detected

Supplementary Table S3. General statistical and model parameters of the multivariate data analysis.

**Parameter**	
Missing values	Random noise imputation within the range of (average noise level – σ ± σ)
Data normalization	UV-scaling
Outlier detection	Hotelling's T^2 (95 %)
7-fold Cross validation	R^2Y (cum)
	Q^2 (cum)
Additional CV	CV-ANOVA
Significant features	95th percentile of features with most characteristic loadings (385 compositions)
Metadata	As given on the beers' label. The UV/Vis measurements were executed for 221 samples as described in Pieczonka et al. (2021), Food Chem., 361 (130112), 1-9
Sample exclusion	For the grain and Purity Law model, 23 samples were excluded due to ambiguous information of the grains used ("rice and/or corn", "cereals")
Statistics software	SIMCA 13.0.3.0 (Umetrics, Umeå, Sweden)

**Statistical model**	**n**	**A**	**R^2Y**	**R^2X**	**Q^2**	**ANOVA**	
						**F**	**p**
Beer type (OPLS-DA)	400	2+5+0	0.865	0.294	0.752	83.27	<< 0.05
Fermentation (OPLS-DA)	400	1+5+0	0.936	0.274	0.792	122.75	<< 0.05
Purity Law (OPLS-DA)	377	1+8+0	0.971	0.325	0.569	26.26	<< 0.05
Grain (OPLS-DA)	377	2+9+0	0.858	0.363	0.579	16.64	<< 0.05
Maillard (OPLS) 294 nm	221	1+3+0	0.941	0.239	0.806	108.60	<< 0.05

Supplementary Table S4. Instrumental parameters and reagents used for FTICR-MS, UPLC-ToF-MS and HPLC-Triple Quad (Folates) measurements.

**Reagent**	**Source**
Methanol (MeOH)	FLUKA, Sigma-Aldrich (LC-MS grade, CHROMASOLV, St Louis, MO, USA)
Acetonitrile (ACN)	FLUKA, Sigma-Aldrich (LC-MS grade, CHROMASOLV, St Louis, MO, USA)
	Honeywell CHROMASOLV® (LC-MS grade)
Ultrapure water	Milli-Q Integral Water Purification System (Millipore, MA, Billerica, USA)
	CHEMSOLUTE® (LC-MS grade)
L-arginine	Sigma-Aldrich (reagent grade >98%, St Louis, MO, USA)
Formic acid (FA)	VWR HiPerSolv CHROMANORM® (LC-MS grade; ≥99%)

**FTICR-MS**	**Value**
Sample preparation	Degassing by ultrasonification (10 °C, 5min.); dilution 1:500 in methanol (v:v); separation of precipitated proteins by centrifugation (10,000 rpm, 3min.)
Spectrometer	Bruker solariX
ICR-cell	Infinity Cell
Direct injection flowrate	120 µL.h^{-1}.
ESI capillary voltage	3600 V (negative ionization) (APOLO II ESI source (Bruker Daltonics GmbH, Bremen, Germany)
Magnetic field	12 T superconducting magnet (Magnex Scientific Inc., Yarton, GB)
Time domain	4 mega words
Accumulation time	0.25 ms
Mass range	*m/z* 120 to 1000
Accumulated scans	400 (for all samples), 2000 (additionally for Samples B1855 and B2019)
Measurement time	10 min.
External calibration	clusters of arginine (5 mg.L^{-1} in methanol)
Internal calibration	in-house calibration list containing 2000 sum formulae, which are highly abundant in beers
Timeframe of analyses	2018 to 2020
Quality Control	Representative beer sample measured as QC within the course of the batches

**UPLC-ToF-MS**	**Value**
Sample preparation	SPE: Bond Elut PPL, 1 mL and 100 mg (Agilent Santa Clara, CA, USA); conditioning: 100 µL MeOH, 2x1000µL Mili-Q-Water + 2% Formic acid (FA); 1000 µL acidified sample (2% FA); washing: 500 µL Mili-Q-Water + 2%FA; dry vacuum; elution: 2x500µL MeOH)
Spectrometer	Shimadzu LCMS-9030 Q ToF
Column	RP (C18: 1.7 µm, 2.1 x 100 mm, Acquity^{TM} UPLC BEH^{TM})
Flow rate	400 µL min^{-1}

Supplementary Table S4 (continued). Instrumental parameters and reagents used for FTICR-MS, UPLC-ToF-MS and HPLC-Triple Quad (Folates) measurements.

UPLC-ToF-MS	Value
ESI ionization mode	negative
Column temperature	40 °C
Injection volume	5 µL (partial loop)
Gradient profile	95 % A (0.1 % formic acid in water) and 5 % B (0.1 % formic acid in acetonitrile) for 1 min; decreasing to 0.5 % A in 5 min; held for 4 min.
Measurement time	10 min.
External calibration	Sodium iodide solution clusters
Internal calibration	ESI-L Low Concentration Tuning Mix
Nitrogen flowrate	10 L min^{-1}
Interface temperature	300°C
Nebulizer gas flow	1 L min^{-1}
Interface voltage	-4 kV
DL temperature	250 °C
Heat block temperature	400 °C
Drying gas flow	10 L min^{-1}
Detector voltage	2 kV
MS^1 parameters	5 Hz event cycle time
	Ion accumulation on
MS^2 fragmentation parameters	DDA (3 dependent events)
	CE spread 20 eV ± 15 eV

UHPLC-Triple Quad-MS (Folates)	Value
Sample preparation	The sample preparation of Striegel et al. (2018), *Front. Chem.*, 6(11) was modified by adding the suitable $^{13}C_5$ labelled isotopologue standard of 10-CHO-PteGlu for quantification. As described in Pferdemenges et al. (2021), *J. Food Compos. Anal.*
Spectrometer	Shimadzu Nexera X2
Column	Raptor™ ARC-18 (2.7 µm, 100 x 2.1 mm)
Precolumn	Raptor™ EXP Guard Column (2.7 µm, 5 x 2.1 mm)
Flow rate	400 µL min^{-1}
Column temperature	30 °C
Injection volume	10 µL
Gradient profile	97 % A (0.1 % formic acid in water) and 3 % B (0.1 % formic acid in acetonitrile) for 1 min; increasing to 10% B in 2 min; held for 2.5 min; increasing to 15 % B in 5 min; increasing to 50 % B in 1 min; held for 1 min; decrease to 3 % B in 1 min; equilibrate for 4 min.
Measurement time	17.5 min
ESI ionization mode	Positive (MRM-multiple reaction monitoring)

Supplementary Table S4 (continued). Instrumental parameters and reagents used for FTICR-MS, UPLC-ToF-MS and HPLC-Triple Quad (Folates) measurements.

**UHPLC-Triple Quad-MS (Folates)**	**Value**
Interface temperature	300 °C
Nebulizer gas flow	3 L min^{-1}
Heating gas flow	10 L min^{-1}
Interface voltage	4 kV
DL temperature	250 °C
Heat block temperature	400 °C
Drying gas flow	10 L min^{-1}
CID gas	270 kPa

Supplementary Table S5. Instrumental parameters, reagents and standards used for NMR-measurements.

Reagent	Source (Purity)
D_2O	Armar chemicals (99.8 atom%, Döttingen, CH)
Di-sodiumhydrogenphosphate dodecahydrate	Merck Millipore (99 %, Billerica, MA, USA)
3-(trimethylsilyl)propionic-2,2,3,3-d4 acid sodium salt	Sigma Aldrich (98 atom%, MO, St Louis, USA)

Standard	Source (Purity)
Furfural	Sigma Aldrich (99 %, MO, St Louis, USA)
Niacin	Sigma Aldrich (≥ 98 %, MO, St Louis, USA)
Histidine	Merck Millipore (≥ 98 %, MA, Billerica, USA)
Xylose	Sigma Aldrich (≥ 99 %, MO, St Louis, USA)

NMR	Value
Spectrometer	800 MHz Bruker AVANCE lll
Probehead	5 mm QCI-probehead
Temperature	300 K
Pusle calibration	Calibrated for each acquisition
Processing	Sine bell with shift 2 in all dimensions
1D-NOE-experiment	
Pulse sequence	90° pulse (12.4 µs), mixing time (100 ms), relaxation delay (16 s), t1 measured by inversion recovery and based on slowest relaxing hydrogen, presaturation for 2.4 sec prior to the first pulse
Cycle time	20 s (AQ = 4 s)
Data acquisition	32 transients, 102562 data points
Spectral width	12,820 Hz
2D-TOCSY (^1H, ^1H)	
Pulse program	dipsi2gpph
Pulse sequence	90° pulse (12.4 µs), mixing time (70 ms), relaxation delay (2.4 s), t1 (42.5 ms), t2 (2 s)
Data acquisition	16 transients, 1024 increments, 48074 data points
Spectral width	12,000 Hz (both dimensions)

Supplementary Table S5. Instrumental parameters, reagents and standards used for NMR-measurements.

NMR	Value
2-D HSQC (^1H, ^{13}C)	
Pulse program	Hsqcetgppssisp2.2
Pulse sequence	90° pulse (^1H 12.4 µs, ^{13}C 11.5 µs), relaxation delay (1.25 s), t1 (3.2 ms), t2 (0.25 s)
Data acquisition	128 transients, 300 increments
Spectral width	12,000 Hz (F2), 46,300 Hz (F1)

Supplementary Table S6. Metadata of the analyzed beer samples.

Sample	Beer style	Fermen-tation	PurityLaw	Grain	Abs. 294 nm	Origin	Sample	Beer style	Fermen-tation	Purity Law	Grain	Abs. 294nm	Origin
**1**	Wheat	top	yes	Wheat	0.048	GER	**238**	Lager	bottom	yes	Barley	0.330	GER
**2**	Lager	bottom	yes	Barley	0.083	GER	**239**	Lager	bottom	no	Rice	-	ESP
**3**	Lager	bottom	yes	Barley	0.105	GER	**241**	Lager	bottom	no	Rice	-	ESP
**4**	Craft	top	no	Barley	0.069	BEL	**242**	Lager	bottom	yes	Barley	0.166	GER
**5**	Wheat	top	yes	Wheat	0.081	GER	**243**	Lager	bottom	yes	Barley	0.133	GER
**6**	Wheat	top	yes	Wheat	0.07	GER	**244**	Lager	bottom	yes	Barley	0.145	GER
**7**	Wheat	top	yes	Wheat	-	GER	**245**	Lager	bottom	yes	Barley	0.115	GER
**8**	Lager	bottom	yes	Barley	0.077	GER	**246**	Lager	bottom	yes	Barley	0.129	GER
**9**	Lager	bottom	yes	Barley	0.162	GER	**247**	Lager	bottom	yes	Barley	0.186	GER
**10**	Lager	bottom	yes	Barley	0.14	GER	**248**	Lager	bottom	yes	Barley	0.111	GER
**11**	Wheat	top	yes	Wheat	-	GER	**249**	Lager	bottom	yes	Barley	0.172	GER
**12**	Wheat	top	yes	Wheat	0.168	GER	**250**	Wheat	top	yes	Wheat	0.158	GER
**13**	Lager	top	yes	Barley	0.17	GER	**251**	Wheat	top	yes	Wheat	0.18	GER
**15**	Lager	bottom	yes	Barley	0.067	GER	**252**	Lager	bottom	yes	Barley	0.129	GER
**16**	Lager	bottom	yes	Barley	0.053	GER	**253**	Wheat	top	yes	Wheat	0.137	GER
**19**	Wheat	top	yes	Wheat	0.136	GER	**254**	Lager	bottom	no	Barley	0.122	CZE
**20**	Wheat	top	yes	Wheat	0.149	GER	**255**	Wheat	top	yes	Wheat	0.159	GER
**21**	Wheat	top	yes	Wheat	0.144	GER	**256**	Wheat	top	yes	Wheat	0.137	GER
**22**	Lager	bottom	yes	Barley	0.138	GER	**257**	Wheat	top	yes	Wheat	0.156	GER
**23**	Lager	bottom	yes	Barley	0.298	GER	**258**	Wheat	top	yes	Wheat	0.185	GER
**24**	Lager	bottom	no	Barley	0.267	IRL	**259**	Wheat	top	yes	Wheat	0.235	GER
**25**	Wheat	top	yes	Wheat	0.2	GER	**260**	Lager	bottom	yes	Barley	0.177	GER
**26**	Lager	bottom	yes	Barley	0.356	GER	**261**	Lager	bottom	yes	Barley	0.167	GER
**27**	Lager	bottom	yes	Barley	0.142	GER	**262**	Lager	bottom	yes	Barley	-	GER
**28**	Wheat	top	yes	Wheat	0.243	GER	**263**	Wheat	top	yes	Wheat	-	GER
**29**	Wheat	top	yes	Wheat	0.224	GER	**264**	Lager	bottom	no	Corn	-	ESP
**30**	Lager	bottom	yes	Barley	0.138	GER	**265**	Lager	bottom	no	Corn	-	ESP
**31**	Wheat	top	yes	Wheat	-	GER	**266**	Lager	bottom	no	Corn	-	ESP
**32**	Craft	top	no	Barley	0.127	BEL	**267**	Lager	bottom	no	Corn	-	ESP
**33**	Wheat	top	yes	Wheat	0.264	GER	**268**	Lager	bottom	no	-	-	ESP
**34**	Wheat	top	yes	Wheat	0.074	GER	**269**	Lager	bottom	no	Corn	-	ESP
**35**	Lager	top	yes	Barley	0.136	GER	**270**	Lager	bottom	no	Corn	-	ESP
**36**	Lager	bottom	yes	Barley	0.126	GER	**271**	Lager	bottom	no	Corn	-	ESP
**37**	Lager	bottom	yes	Barley	0.42	GER	**272**	Lager	bottom	no	Rice	0.358	ESP
**38**	Wheat	top	yes	Wheat	0.124	GER	**273**	Craft	top	yes	Barley	-	ESP
**39**	Lager	bottom	yes	Barley	0.145	GER	**274**	Lager	bottom	no	Rice	-	ESP
**41**	Wheat	top	yes	Wheat	0.139	GER	**275**	Lager	bottom	no	Corn	0.139	ESP
**43**	Craft	top	yes	Barley	0.129	BEL	**276**	Lager	bottom	no	Corn	-	ESP
**44**	Lager	bottom	yes	Barley	0.122	GER	**277**	Lager	bottom	no	Corn	-	ESP
**45**	Wheat	top	yes	Wheat	0.13	GER	**278**	Craft	top	yes	Barley	-	ESP
**46**	Lager	bottom	yes	Barley	0.099	GER	**279**	Lager	bottom	yes	Barley	-	ESP
**47**	Lager	bottom	yes	Wheat	0.113	GER	**280**	Lager	bottom	no	Corn	-	ESP
**48**	Lager	bottom	yes	Barley	0.399	GER	**281**	Craft	top	yes	Barley	-	ESP
**49**	Craft	top	yes	Barley	0.203	GER	**282**	Craft	top	yes	Barley	0.386	ESP
**50**	Lager	bottom	yes	Barley	0.121	GER	**283**	Lager	bottom	no	Corn	-	ESP
**51**	Wheat	top	yes	Wheat	0.197	GER	**284**	Craft	top	yes	Barley	0.222	ESP
**52**	Wheat	top	yes	Wheat	0.149	GER	**285**	Lager	bottom	no	Corn	0.16	ESP
**53**	Lager	bottom	yes	Barley	-	GER	**286**	Craft	top	yes	Barley	-	ESP
**54**	Craft	top	no	Barley	-	BEL	**287**	Craft	top	yes	Barley	-	ESP
**55**	Craft	top	no	Wheat	0.187	LTU	**288**	Craft	top	no	Barley	-	BEL
**56**	Wheat	top	yes	Wheat	0.169	GER	**289**	Craft	top	no	Wheat	-	BEL
**57**	Craft	top	yes	Barley	0.421	GER	**290**	Wheat	top	no	Wheat	0.169	NLD
**58**	Wheat	top	yes	Wheat	0.156	GER	**291**	Lager	top	no	Barley	-	BEL
**59**	Craft	top	no	Barley	-	BEL	**292**	Craft	top	no	Barley	0.13	BEL
**60**	Craft	top	yes	Barley	0.282	GER	**293**	Lager	bottom	yes	Barley	0.27	HRV
**61**	Craft	top	yes	Wheat	0.191	GER	**294**	Lager	bottom	yes	Barley	-	SLO
**62**	Craft	top	yes	Wheat	-	GER	**295**	Lager	bottom	yes	Barley	-	HRV
**63**	Wheat	top	yes	Wheat	-	GER	**296**	Lager	bottom	no	Corn	-	HRV
**64**	Craft	top	yes	Barley	0.174	GER	**297**	Lager	bottom	yes	-	-	HRV
**65**	Craft	top	no	Barley	0.296	BEL	**298**	Craft	top	yes	Barley	-	HRV
**66**	Craft	top	no	Barley	0.33	BEL	**299**	Craft	top	yes	Wheat	-	HRV
**67**	Wheat	top	no	Wheat	0.167	GER	**300**	Lager	bottom	yes	Barley	0.252	HRV
**68**	Lager	bottom	yes	Barley	0.218	GER	**301**	Craft	top	yes	Barley	-	HRV
**69**	Wheat	top	no	Wheat	0.095	BEL	**302**	Lager	bottom	no	Barley	-	HRV
**70**	Lager	bottom	yes	Barley	0.048	NAM	**303**	Wheat	top	yes	Wheat	-	HRV
**71**	Lager	bottom	yes	Barley	-	DNK	**304**	Lager	bottom	yes	Barley	-	HRV
**72**	Lager	bottom	yes	Barley	-	GER	**305**	Wheat	top	yes	Wheat	-	HRV
**73**	Lager	bottom	yes	Barley	-	GER	**306**	Lager	bottom	yes	Barley	-	JPN
**76**	Wheat	top	yes	Wheat	0.279	GER	**307**	Lager	bottom	no	Rice	-	JPN
**78**	Craft	top	yes	Barley	0.275	GER	**308**	Lager	top	yes	Wheat	-	JPN
**80**	Lager	bottom	no	Rice	0.112	GER	**309**	Lager	bottom	no	Rice	-	JPN
**81**	Craft	top	yes	Barley	0.222	GER	**310**	Craft	top	yes	Barley	-	USA
**83**	Lager	bottom	yes	Barley	0.243	GER	**311**	Lager	bottom	no	Rice	-	JPN
**85**	Craft	top	yes	Wheat	-	GER	**312**	Lager	bottom	no	Rice	-	JPN
**86**	Craft	top	yes	Barley	0.238	GER	**313**	Lager	bottom	no	Rice	-	JPN
**90**	Craft	top	yes	Barley	0.136	GER	**314**	Wheat	top	no	Wheat	-	FRA
**91**	Lager	bottom	no	Barley	0.099	CUB	**315**	Lager	top	no	Barley	-	FRA
**92**	Lager	bottom	no	Barley	0.063	CUB	**317**	Lager	bottom	no	-	-	ESP
**93**	Lager	bottom	yes	Barley	0.232	MEX	**318**	Lager	bottom	no	Rice	-	ESP
**94**	Lager	bottom	yes	Barley	0.134	MEX	**319**	Lager	bottom	no	Corn	-	ESP
**95**	Lager	bottom	no	Rice	0.086	CHN	**320**	Lager	bottom	no	Corn	-	ESP
**96**	Lager	bottom	yes	Barley	0.16	PER	**321**	Lager	bottom	no	Rice	-	ESP
**97**	Lager	bottom	no	Corn	0.1	ARG	**322**	Lager	bottom	no	-	-	ESP
**98**	Lager	bottom	yes	Barley	-	PER	**323**	Lager	bottom	no	Corn	-	ESP

Supplementary Table S6 (continued). Metadata of the analyzed beer samples.

Sample	Beer style	Fermen -tation	Purity Law	Grain	Abs. 294 nm	Origin	Sample	Beer style	Fermen -tation	Purity Law	Grain	Abs. 294nm	Origin
**99**	Lager	bottom	no	Barley	0.137	ESP	**324**	Lager	bottom	no	Rice	-	ESP
**100**	Craft	top	no	-	-	BRA	**325**	Lager	bottom	no	-	-	ESP
**101**	Craft	top	no	Rice	0.189	JPN	**326**	Lager	bottom	yes	Barley	-	ESP
**102**	Wheat	top	no	Wheat	0.116	NDL	**327**	Lager	bottom	no	Rice	-	ESP
**103**	Lager	bottom	no	Barley	0.104	KOR	**328**	Lager	bottom	no	Corn	-	ESP
**104**	Craft	top	yes	Barley	-	GER	**329**	Lager	bottom	no	Corn	-	ESP
**105**	Craft	top	no	Wheat	0.121	BEL	**330**	Lager	bottom	no	Corn	-	ESP
**106**	Craft	top	yes	Wheat	0.36	BEL	**331**	Lager	bottom	no	Rice	-	ESP
**107**	Craft	top	no	Barley	0.192	BEL	**333**	Lager	bottom	yes	Barley	-	ESP
**108**	Craft	top	no	Wheat	0.107	BEL	**334**	Lager	bottom	no	Rice	-	PHL
**109**	Craft	top	yes	Barley	0.368	BEL	**335**	Lager	bottom	no	Rice	-	THA
**110**	Craft	top	yes	Barley	0.204	NDL	**336**	Lager	bottom	no	Rice	-	TWN
**111**	Lager	bottom	yes	Barley	0.134	NDL	**337**	Lager	bottom	no	Rice	-	SGP
**112**	Craft	top	yes	Barley	0.219	NDL	**357**	Craft	top	yes	Wheat	-	GER
**113**	Lager	bottom	yes	Barley	0.167	GER	**358**	Lager	bottom	yes	Barley	-	GER
**114**	Lager	bottom	yes	Barley	0.127	SGP	**360**	Craft	top	no	-	-	MEX
**116**	Craft	top	no	Barley	0.238	BEL	**361**	Lager	bottom	no	-	-	MEX
**117**	Craft	top	yes	Wheat	0.167	BEL	**362**	Lager	bottom	no	-	-	MEX
**118**	Craft	top	yes	Wheat	-	BEL	**363**	Lager	bottom	yes	-	-	MEX
**119**	Craft	top	yes	Barley	0.709	NDL	**364**	Craft	top	no	Wheat	-	ZAF
**121**	Craft	top	yes	Wheat	-	BEL	**365**	Craft	top	no	Barley	-	USA
**123**	Craft	top	no	Wheat	0.132	NDL	**366**	Lager	bottom	no	-	-	EGY
**124**	Craft	top	yes	Wheat	0.418	GER	**367**	Wheat	top	yes	Wheat	-	NAM
**125**	Craft	top	no	Barley	0.276	BEL	**368**	Craft	top	yes	Barley	-	ZAF
**126**	Craft	top	yes	Wheat	-	BEL	**369**	Lager	bottom	yes	Barley	-	ZAF
**127**	Lager	bottom	yes	Barley	0.334	GER	**370**	Craft	top	yes	Barley	-	USA
**128**	Craft	top	yes	Barley	0.657	GER	**371**	Wheat	top	yes	Wheat	-	GER
**129**	Lager	bottom	yes	Barley	0.197	NDL	**372**	Wheat	top	yes	Wheat	-	ZAF
**130**	Craft	top	no	Barley	0.353	BEL	**373**	Lager	bottom	no	-	-	ARG
**131**	Craft	top	yes	Barley	0.144	BEL	**374**	Lager	bottom	no	-	-	ARG
**132**	Craft	top	no	Barley	0.183	BEL	**375**	Lager	bottom	yes	Barley	-	ZAF
**133**	Craft	top	no	Wheat	0.187	BEL	**376**	Lager	bottom	yes	Barley	-	BRA
**134**	Craft	top	no	Barley	-	BEL	**377**	Lager	bottom	no	-	-	BRA
**136**	Lager	bottom	yes	Barley	0.095	FRA	**378**	Lager	bottom	no	-	-	MEX
**137**	Wheat	top	yes	Wheat	-	GER	**379**	Lager	bottom	no	Corn	-	MEX
**138**	Craft	top	no	Wheat	0.076	BEL	**380**	Craft	top	yes	Barley	-	USA
**139**	Lager	bottom	no	Barley	0.128	IND	**381**	Lager	bottom	no	Rice	-	JPN
**140**	Lager	bottom	yes	Barley	0.288	GER	**382**	Lager	bottom	no	Rice	-	CHN
**141**	Lager	bottom	yes	Barley	0.409	GER	**383**	Lager	bottom	no	Corn	-	BRA
**142**	Lager	bottom	yes	Barley	0.177	GER	**385**	Lager	bottom	yes	Barley	-	CHE
**143**	Lager	bottom	yes	Barley	0.137	GER	**386**	Craft	top	yes	-	-	CHN
**144**	Lager	bottom	yes	Barley	0.086	GER	**387**	Craft	top	yes	Barley	-	USA
**145**	Lager	bottom	no	Barley	0.102	MEX	**388**	Lager	bottom	no	-	-	BRA
**146**	Lager	bottom	yes	Barley	0.095	BEL	**389**	Craft	top	no	Barley	0.627	ESP
**147**	Lager	bottom	yes	Barley	0.326	GER	**390**	Craft	top	no	Barley	-	ESP
**148**	Lager	bottom	yes	Barley	0.153	GER	**391**	Lager	bottom	no	Rice	-	MEX
**149**	Craft	top	no	Barley	0.19	BEL	**392**	Lager	bottom	yes	Barley	-	FRA
**151**	Craft	top	no	Corn	0.189	FRA	**393**	Craft	top	yes	Barley	-	ITA
**152**	Wheat	top	yes	Wheat	0.118	GER	**394**	Craft	top	no	Rice	0.709	ITA
**153**	Lager	bottom	yes	Barley	0.147	GER	**395**	Craft	top	no	Wheat	-	ITA
**154**	Lager	bottom	yes	Barley	0.146	GER	**396**	Craft	top	yes	Barley	-	ITA
**155**	Wheat	top	yes	Wheat	0.349	GER	**397**	Craft	top	yes	Wheat	-	ITA
**156**	Craft	top	no	Corn	0.287	FRA	**398**	Craft	top	yes	Barley	0.46	ESP
**157**	Lager	bottom	yes	Barley	0.184	GER	**399**	Craft	top	yes	Barley	-	CRO
**159**	Lager	bottom	yes	Barley	0.123	GER	**400**	Craft	top	no	Wheat	-	ESP
**160**	Lager	bottom	no	Corn	-	CHN	**401**	Craft	top	yes	Barley	-	GRE
**161**	Lager	bottom	no	-	0.118	ITA	**402**	Craft	top	no	Wheat	-	EST
**162**	Lager	bottom	no	Barley	0.12	PHL	**404**	Craft	top	no	Wheat	-	GRE
**163**	Lager	bottom	yes	Barley	0.112	GER	**405**	Craft	top	no	Wheat	-	CAT
**164**	Lager	bottom	no	-	0.115	GBR	**406**	Craft	top	yes	Barley	-	POR
**165**	Lager	bottom	yes	Barley	0.137	GER	**407**	Lager	bottom	no	Rice	-	CHN
**166**	Lager	bottom	no	Rice	0.126	THA	**408**	Lager	bottom	no	-	-	ITA
**167**	Lager	bottom	no	Corn	0.124	ITA	**409**	Craft	top	no	Wheat	-	FRA
**168**	Lager	bottom	no	Rice	0.114	CHN	**410**	Lager	bottom	no	Rice	-	VAT
**169**	Lager	bottom	no	Corn	0.132	ITA	**411**	Lager	bottom	no	Corn	-	CHN
**170**	Lager	bottom	no	Rice	0.088	GTM	**412**	Lager	bottom	no	Wheat	-	NLD
**171**	Lager	bottom	no	Rice	0.088	GTM	**413**	Lager	bottom	no	Rice	-	CHN
**172**	Lager	bottom	no	Corn	0.089	CRI	**414**	Lager	bottom	yes	Barley	-	GER
**173**	Lager	bottom	no	Rice	0.084	NIC	**416**	Craft	top	yes	Barley	0.247	GER
**174**	Craft	top	yes	Barley	0.153	FIN	**417**	Craft	top	yes	Barley	0.685	NDL
**175**	Lager	bottom	no	Barley	0.103	FIN	**418**	Craft	top	yes	Barley	0.456	GBR
**177**	Craft	top	yes	Barley	0.331	FIN	**419**	Craft	top	yes	Barley	0.457	GBR
**178**	Lager	bottom	yes	Barley	0.108	LUX	**420**	Craft	top	no	Barley	0.367	GER
**179**	Lager	bottom	yes	Barley	0.123	FRA	**421**	Craft	top	no	Barley	0.435	GBR
**180**	Lager	bottom	yes	Barley	0.188	LUX	**422**	Craft	top	no	Barley	0.613	GER
**182**	Craft	top	yes	Barley	0.183	NZL	**424**	Craft	top	no	Barley	0.687	BEL
**191**	Lager	bottom	no	Corn	0.135	BEL	**425**	Craft	top	no	Barley	0.432	GBR
**192**	Lager	bottom	yes	Barley	-	GER	**426**	Craft	top	yes	Barley	-	GER
**193**	Lager	bottom	yes	Barley	0.121	GER	**427**	Craft	top	yes	Barley	0.804	GER
**194**	Lager	bottom	yes	Wheat	0.525	GER	**428**	Craft	top	yes	Barley	0.196	GER
**197**	Craft	top	no	Barley	0.262	GER	**429**	Lager	bottom	yes	Barley	0.18	GER
**198**	Craft	top	no	Wheat	-	POL	**430**	Craft	top	yes	Barley	0.393	GBR
**199**	Craft	top	no	Barley	-	POL	**431**	Craft	top	yes	Barley	0.466	BEL
**200**	Wheat	top	yes	Wheat	0.152	GER	**432**	Craft	top	yes	Barley	0.679	GER
**201**	Lager	bottom	yes	Barley	0.098	CHE	**433**	Lager	bottom	yes	Barley	-	GER

Supplementary Table S6 (continued). Metadata of the analyzed beer samples.

Sample	Beer style	Fermen -tation	Purity Law	Grain	Abs. 294 nm	Origin	Sample	Beer style	Fermen -tation	Purity Law	Grain	Abs. 294nm	Origin
**202**	Craft	top	no	Wheat	0.155	POL	**434**	Lager	bottom	yes	Barley	-	GER
**203**	Lager	bottom	yes	Barley	0.168	ZAF	**435**	Lager	bottom	yes	Barley	-	GER
**205**	Lager	bottom	yes	Barley	0.127	CHE	**436**	Lager	bottom	yes	Barley	-	GER
**206**	Lager	bottom	no	Barley	0.966	POL	**437**	Craft	top	no	Rice	-	BEL
**207**	Craft	top	no	Wheat	0.781	POL	**438**	Craft	top	yes	Barley	-	BEL
**208**	Lager	bottom	yes	Barley	0.163	GER	**439**	Craft	top	no	Wheat	-	BEL
**209**	Lager	bottom	yes	Barley	0.193	POL	**440**	Craft	top	yes	Barley	-	NLD
**210**	Craft	top	no	Barley	-	POL	**441**	Craft	top	no	Wheat	-	NLD
**211**	Craft	top	yes	Barley	0.89	POL	**442**	Wheat	top	no	Wheat	-	NLD
**212**	Craft	top	yes	Barley	0.155	HUN	**443**	Lager	bottom	yes	Barley	-	NLD
**213**	Lager	bottom	no	Rice	0.145	ESP	**444**	Lager	bottom	yes	Barley	-	BEL
**214**	Lager	bottom	yes	Barley	0.168	GER	**445**	Craft	top	yes	Barley	-	NLD
**215**	Craft	top	yes	Barley	0.185	GER	**446**	Craft	top	no	Wheat	-	BEL
**216**	Lager	bottom	yes	Barley	0.157	GER	**447**	Craft	top	yes	Barley	-	GER
**217**	Wheat	top	yes	Wheat	0.249	GER	**448**	Craft	top	yes	Barley	-	NLD
**218**	Craft	top	yes	Barley	0.134	GER	**449**	Craft	top	no	Wheat	-	BEL
**219**	Craft	top	yes	Barley	0.27	GER	**450**	Lager	bottom	yes	Wheat	-	GER
**220**	Craft	top	yes	Barley	0.208	GER	**451**	Craft	top	no	Barley	-	BEL
**221**	Craft	top	yes	Wheat	0.132	GER	**452**	Craft	top	no	Rice	-	BEL
**222**	Lager	bottom	no	-	0.114	JPN	**453**	Wheat	top	yes	Wheat	-	GER
**223**	Lager	bottom	yes	Barley	0.569	GER	**454**	Craft	top	no	Barley	-	BEL
**224**	Lager	bottom	yes	Barley	0.211	GER	**455**	Lager	bottom	no	Barley	-	IRL
**226**	Lager	bottom	yes	Barley	0.238	GER	**456**	Craft	top	no	Barley	-	BEL
**227**	Wheat	top	yes	Wheat	0.207	GER	**457**	Lager	bottom	yes	Barley	-	GER
**228**	Lager	bottom	no	Corn	0.14	ESP	**458**	Lager	bottom	yes	Barley	-	GER
**229**	Lager	bottom	no	Corn	0.158	ESP	**459**	Lager	bottom	yes	Barley	-	ISR
**230**	Lager	bottom	no	Corn	0.143	ESP	**460**	Lager	bottom	yes	-	-	ISR
**231**	Lager	bottom	no	Corn	0.096	ESP	**464**	Lager	bottom	no	Rice	-	THA
**232**	Lager	bottom	no	-	0.193	ESP	**465**	Lager	bottom	no	Rice	-	THA
**235**	Lager	bottom	no	Corn	-	ESP	**467**	Lager	bottom	no	Rice	-	THA
**236**	Lager	bottom	no	Corn	-	ESP	**B1885**	-	-	-	-	-	GER
**237**	Lager	bottom	no	Corn	-	ESP	**B2019**	Lager	bottom	no	Barley	-	GER

Supplementary Table S7. Score values of the samples.

Sample	Beer style		Fermentation		Purity Law		Grain			Abs. 294nm	
	PC1	PC2	PC1	PC2	PC1	PC2	PC1	PC2	PC3	PC1	PC2
**1**	-2.49	18.71	8.09	19.04	10.21	37.73	-10.17	-15.51	-2.89	-23.81	-27.70
**2**	-12.73	-3.39	-10.99	12.35	9.92	14.49	12.52	3.04	-3.47	-21.97	8.11
**3**	-12.17	-4.88	-10.78	10.09	8.98	12.79	12.51	2.05	-3.08	-18.21	7.10
**4**	7.31	-1.20	9.23	22.08	-11.14	22.15	6.82	3.37	-2.66	-15.56	-11.13
**5**	-4.45	38.37	15.09	20.97	9.73	17.99	-9.66	-21.02	1.51	-19.03	2.17
**6**	-0.45	37.37	16.56	27.06	8.07	20.35	-12.96	-19.25	1.08	-19.08	-4.65
**7**	0.23	33.60	12.43	16.11	6.86	6.03	-13.62	-15.55	-1.76	-	-
**8**	-11.47	-0.62	-9.81	8.70	10.17	10.93	12.91	4.77	-4.67	-12.76	7.19
**9**	-11.93	-1.82	-7.94	12.82	8.74	8.83	13.78	4.32	-4.56	-14.46	15.44
**10**	-6.12	-5.86	-5.67	16.65	11.82	22.02	13.24	0.79	1.53	-16.11	-3.10
**11**	-4.87	16.82	7.72	24.42	6.92	56.56	-11.85	-15.11	-3.19	-	-
**12**	-3.01	22.54	9.88	31.99	7.25	43.59	-6.94	-12.22	0.18	-12.31	-34.56
**13**	-3.71	-0.12	8.86	13.28	7.1	5.14	10.69	2.44	-1.17	-6.44	12.25
**15**	-11.67	-3.13	-11.46	14.76	10.72	35.14	9.63	0.54	-2.98	-14.69	-25.25
**16**	-8.58	-3.32	-8.04	13.01	10.81	23.79	13.28	-0.06	-1.19	-15.27	-11.26
**19**	-2.69	30.88	13.74	22.81	12.02	22.16	-8.94	-20.70	-1.25	-16.06	-9.15
**20**	-2.83	19.92	7.45	26.28	8.81	41.92	-8.29	-13.93	-0.91	-9.33	-37.09
**21**	-4.69	16.65	4.44	29.15	8.31	48.9	-5.72	-11.01	-0.84	-10.77	-44.02
**22**	-9.52	0.92	-9.08	21.46	6.83	46.27	5.59	2.60	-1.54	-8.76	-40.99
**23**	-8.54	0.15	-9.53	20.43	8.83	37.36	10.77	2.02	-1.73	5.03	-32.73
**24**	-2.70	0.61	-4.22	18.73	-12.6	10.23	6.21	0.44	-0.81	4.31	-41.97
**25**	1.37	14.36	8.32	17.12	8.91	36.83	-8.52	-9.14	-0.51	-2.84	-35.34
**26**	-7.95	2.08	-7.10	-5.85	9.56	14.23	11.81	2.94	0.07	15.86	-14.37
**27**	-10.64	-0.43	-10.67	17.31	10.49	40.15	11.68	1.50	-0.93	-13.04	-30.83
**28**	-0.74	15.59	7.07	29.31	8.29	49.96	-3.74	-10.63	-0.47	-5.76	-49.19
**29**	3.23	19.77	11.63	27.09	8.2	40.03	-6.59	-16.52	2.29	-2.76	-40.17
**30**	-7.55	0.74	-6.40	23.19	7.84	44.88	7.55	0.96	-0.91	-8.95	-39.33
**31**	-3.77	19.29	5.48	22.72	7.86	42.17	-5.18	-12.49	-0.94	-	-
**32**	7.89	-0.18	8.09	12.46	-10.38	15.85	1.61	2.12	-2.80	-1.88	-15.79
**33**	4.99	26.91	18.02	9.88	9	3.35	-8.42	-18.95	1.37	2.32	0.87
**34**	1.49	26.47	14.26	25.64	7.94	31.34	-8.56	-15.34	0.73	-15.55	-23.69
**35**	-1.97	-3.74	6.78	12.85	6.38	3.7	11.10	1.40	-1.66	-15.67	11.34
**36**	-7.11	-1.89	-6.28	15.89	7.49	30.83	11.28	-0.62	0.93	-13.08	-22.63
**37**	-9.70	-4.30	-9.55	-43.99	8.86	-22.05	11.82	0.07	-3.80	26.69	14.41
**38**	-4.10	21.09	8.05	16.41	9.85	28.66	-4.43	-14.77	0.92	-7.65	-23.43
**39**	-13.38	0.77	-11.25	13.37	9.72	32.69	8.18	0.82	-2.30	-9.73	-23.64
**41**	2.66	23.31	12.59	21.74	6.61	28.66	-8.66	-15.30	-1.60	-9.36	-26.09
**43**	5.53	2.23	8.31	22.83	8.67	-23.58	1.12	0.01	-1.47	-10.17	-22.29
**44**	-9.24	-0.42	-8.54	16.77	10.45	39.04	10.74	-0.49	-0.77	-13.01	-31.16
**45**	-2.76	25.17	10.93	23.33	9.5	29.8	-5.70	-16.65	0.41	-13.30	-21.83
**46**	-4.10	0.60	-7.18	19.67	8.26	54.12	4.31	-2.46	-1.22	-14.38	-51.72
**47**	0.27	1.67	-2.86	19.98	7.59	54.02	-1.26	-9.82	-1.79	-12.16	-54.36
**48**	-5.43	4.40	-5.55	1.98	8.28	30.96	5.37	-0.88	0.19	12.43	-36.68
**49**	12.35	-4.37	9.19	25.73	7.98	39.96	6.75	-0.21	0.41	-5.39	-37.84
**50**	-8.37	-0.53	-6.27	20.81	8.49	33.3	11.93	4.27	-0.35	-14.11	-17.22
**51**	-4.16	30.37	11.54	24.81	6.93	25.99	-11.96	-15.43	-0.48	-10.74	-13.69
**52**	-0.31	30.03	14.26	25.42	8.21	25.46	-10.10	-17.32	1.61	-9.98	-17.49
**53**	-12.59	-7.15	-9.17	5.91	9.9	-17.51	10.06	2.68	-4.19	-	-
**54**	12.35	4.06	9.89	17.73	-6.77	37.4	1.92	-2.42	-0.51	-	-
**55**	16.90	-3.53	12.23	28.42	-13.1	-12.56	1.71	-10.12	0.45	-4.31	-51.46
**56**	-3.20	29.92	12.31	16.32	11.21	15.23	-7.74	-17.72	-2.61	-11.90	-2.91
**57**	20.63	-8.00	17.04	-0.56	7.75	-8.37	8.26	0.26	0.81	24.15	2.90
**58**	-1.36	21.58	10.60	24.09	6.97	29.61	-6.63	-16.50	0.35	-12.79	-22.32
**59**	19.47	-4.47	11.01	6.11	-12.62	20.23	3.16	0.01	-1.76	-	-
**60**	16.95	-10.89	13.97	5.44	10.84	-4.37	11.52	1.58	-1.34	7.90	7.91
**61**	12.47	-2.92	8.22	14.03	10.13	37.72	-3.40	-13.26	-2.84	-5.00	-37.91
**62**	17.28	2.41	13.95	15.51	9.8	16.62	-9.37	-18.90	-2.35	-	-
**63**	1.95	38.48	8.99	19.05	8.6	2	-8.04	-21.04	3.15	-	-
**64**	10.88	-5.03	9.99	16.79	9.15	17.82	11.89	-1.60	1.09	-6.50	-11.08
**65**	22.76	-5.07	18.52	19.54	-13.98	10.38	9.29	-0.12	0.73	7.67	-13.49
**66**	21.18	-2.42	16.33	20.51	-9.03	31.95	9.30	2.13	-0.63	16.03	-41.61
**67**	2.54	18.29	11.88	17.72	-13.96	5.19	-6.47	-15.98	3.09	-11.22	-13.44
**68**	-7.48	4.30	-8.37	-2.92	7.84	26	5.74	0.03	-0.18	10.33	-30.25
**69**	6.32	12.31	13.12	21.92	-5.89	17.11	-3.11	-11.65	0.29	-11.67	-12.10
**70**	-8.38	-0.90	-6.92	17.51	10.2	29.75	13.47	-0.21	0.69	-16.96	-17.33
**71**	-9.69	0.03	-7.87	15.80	5.01	28.84	7.36	3.29	1.51	-	-
**72**	-13.46	-0.58	-12.35	16.73	7.22	35.93	7.49	3.97	-1.99	-	-
**73**	-11.69	-0.29	-11.41	12.10	9.22	35.9	6.77	-0.09	0.13	-	-
**76**	2.47	23.28	13.95	20.77	6.94	27.51	-6.77	-17.28	0.80	-1.61	-25.14
**78**	15.57	-4.52	12.33	13.58	8.83	18.92	8.18	1.00	3.24	7.10	-19.26
**80**	-6.51	1.53	-8.55	22.47	-8.81	41.04	-6.24	9.88	5.59	-13.73	-34.93
**81**	18.50	-5.15	13.07	22.02	7.33	32.54	3.03	-1.42	0.85	0.29	-35.04
**83**	-0.91	-1.04	-5.08	7.10	9.22	25.71	7.43	0.08	0.52	0.66	-23.88
**85**	8.48	1.52	8.58	20.20	8.19	36.46	-2.96	-8.99	-0.42	-	-
**86**	12.85	-6.87	9.67	24.77	10.41	33.05	11.70	-0.15	0.00	-4.88	-29.43
**90**	21.13	-8.34	15.99	5.82	7.87	22.74	11.99	0.92	-5.30	-7.23	-12.27
**91**	-9.52	-5.74	-9.07	-20.94	-11.07	-54.1	11.55	2.63	-5.10	-11.47	91.02
**92**	-12.89	-5.46	-11.22	-16.75	-12.74	-44.83	10.77	1.89	-0.03	-13.31	83.30
**93**	-17.47	-4.68	-13.26	-75.35	9.65	-87.94	6.00	3.35	-2.48	4.71	115.1
**94**	-13.40	-1.88	-10.28	-23.77	8.87	-53.57	3.62	0.19	-1.35	-10.99	98.66
**95**	-16.81	-4.29	-14.50	-13.73	-12.55	-29.81	-14.49	15.68	21.56	-18.96	62.05
**96**	-11.74	-8.64	-10.40	-19.03	9.06	-52.37	9.06	0.08	-2.87	-4.52	97.57
**97**	-9.67	-4.26	-11.80	-9.41	-12.01	-31.78	-24.39	13.00	-14.70	-13.51	59.39

Supplementary table S7 (continued). Score values of the samples.

Sample	Beer style		Fermentation		Purity Law		Grain			Abs. 294nm	
	PC1	PC2	PC1	PC2	PC1	PC2	PC1	PC2	PC3	PC1	PC2
**98**	-6.23	-4.21	-11.15	-32.93	9.65	-11.11	6.07	1.04	-2.13	-	-
**99**	-21.19	-1.34	-20.06	-40.91	-10.62	-96.16	13.66	8.35	-0.52	-22.13	120.4
**100**	21.90	-5.67	13.86	19.87	-11.28	36.31	-	-	-	-	-
**101**	23.45	-4.20	14.83	-26.95	-11.26	-81.44	-18.30	18.25	26.14	-11.44	95.13
**102**	5.70	23.88	14.56	13.88	-11.88	-17.9	-13.43	-19.55	1.58	-23.96	33.81
**103**	-7.59	-5.73	-12.96	11.03	-13.21	-11.83	10.37	-1.35	-2.45	-18.51	41.82
**104**	23.15	-8.47	16.18	8.85	6.03	27.6	11.51	2.16	-6.88	-	-
**105**	20.77	5.00	16.62	20.73	-14.26	-2.84	-7.88	-17.88	-0.44	-15.50	12.74
**106**	21.41	-2.24	13.30	-46.70	11	-44.98	-7.41	-16.59	-1.48	23.71	30.93
**107**	14.14	-7.99	13.14	4.18	-12.5	-16.67	8.99	1.26	-2.46	-2.53	28.73
**108**	20.62	9.49	17.98	20.29	-11.49	-8.57	-9.76	-18.63	1.97	-19.78	22.44
**109**	20.27	-8.74	11.81	-38.07	9.24	-26.19	7.39	2.91	-2.80	30.66	8.53
**110**	14.91	-10.65	12.05	-6.72	8.2	-28.12	9.67	-2.63	0.83	-3.80	39.01
**111**	-10.62	-6.53	-10.85	-8.40	7.64	-26.6	12.12	-0.35	-4.84	-16.64	55.60
**112**	21.24	-11.42	14.68	4.49	9.23	-14.94	6.44	0.04	3.26	-1.97	24.84
**113**	-9.09	-6.27	-10.10	-19.08	9.2	-21.14	9.44	-2.37	1.11	-6.36	33.97
**114**	-9.41	-5.82	-9.82	10.38	6.59	20.65	3.20	3.05	-1.83	-13.86	-0.22
**116**	10.96	-4.08	11.34	9.72	-9.74	3.61	8.62	-0.29	-1.57	0.21	1.68
**117**	22.81	-2.24	14.00	12.41	8.68	17.16	-9.03	-18.22	1.82	-8.82	13.30
**118**	19.85	-2.24	11.02	22.26	10.26	25.39	-8.42	-15.82	1.77	-	-
**119**	23.64	-7.28	15.28	-51.34	10.7	-39.59	5.09	-0.66	-0.43	65.38	6.25
**121**	22.04	-4.52	13.03	8.73	7.44	24.18	-7.53	-11.73	-1.18	-	-
**123**	10.61	-6.65	8.18	8.02	-11.82	-0.15	-3.10	-7.00	-0.23	-10.17	3.90
**124**	13.98	4.70	12.53	-46.04	9.23	-29.77	-7.25	-13.71	-0.73	42.16	3.04
**125**	25.04	-6.73	19.27	15.39	-13.35	-1.87	13.47	1.49	2.76	7.81	2.60
**126**	25.91	-7.60	12.24	-2.90	10.37	10.18	-7.98	-16.55	-0.03	-	-
**127**	-7.66	-7.68	-11.38	-59.61	8.6	-54.21	11.73	2.54	-2.62	18.61	51.27
**128**	18.38	-4.93	18.46	-	8.6	-	11.24	-2.33	0.81	59.31	63.90
**129**	-4.21	-1.11	-9.26	-3.63	11.74	23.02	9.33	-3.86	-1.26	-4.02	-17.39
**130**	25.35	-3.73	14.98	-8.19	-12.03	-5.14	9.75	1.07	-0.19	25.45	-13.53
**131**	11.93	-4.37	9.16	14.54	7.93	-31.16	4.16	-1.38	2.16	-8.50	-8.64
**132**	20.36	-8.44	13.77	5.28	-14.2	-0.56	10.36	0.48	1.06	0.54	-4.60
**133**	22.58	0.29	18.52	16.60	-9.59	-1.13	-1.99	-16.74	4.15	-6.35	1.14
**134**	27.70	-10.25	13.69	-15.80	-13.88	-26.37	12.90	4.48	-4.32	-	-
**136**	-10.51	-8.68	-12.67	11.12	9.86	13.22	5.58	2.42	-5.36	-11.34	10.41
**137**	10.40	28.94	15.78	-	8.32	-124.5	-5.15	-17.09	-3.08	-	-
**138**	12.32	5.29	11.82	25.05	-11.27	14.21	-4.78	-16.36	3.84	-19.21	1.70
**139**	-17.01	-7.52	-13.57	-24.49	-14.69	-28.51	13.74	-3.22	-0.44	-11.68	50.97
**140**	-8.56	-4.24	-10.70	-26.77	8.17	-16.66	14.51	1.68	-2.39	12.70	29.44
**141**	-18.12	-7.64	-17.34	-75.98	5.72	-50.19	11.58	0.72	-5.31	26.87	47.36
**142**	-9.20	-2.81	-8.28	-9.62	5.77	7.21	10.11	2.96	1.34	7.39	-0.99
**143**	-13.07	-5.51	-12.07	15.84	11.82	20.39	15.90	0.31	-1.23	-8.98	6.68
**144**	-15.29	-4.86	-14.69	13.60	7.36	32.13	10.23	3.85	-0.46	-14.60	-18.97
**145**	-11.92	-4.66	-13.46	11.93	-12.52	21.61	4.53	2.71	1.64	-11.46	-14.60
**146**	-6.93	-5.35	-9.40	17.72	7.62	-9.68	6.76	1.44	-0.38	-18.19	-7.67
**147**	-9.96	-1.97	-9.49	-29.86	8.67	-13.9	10.92	0.71	-2.59	17.54	14.65
**148**	-13.71	-3.42	-12.43	2.48	8.9	10.86	9.12	0.00	-1.85	-10.45	3.31
**149**	18.16	-6.68	14.70	20.24	-12.11	22.18	4.98	-1.50	-1.68	-0.74	-25.19
**151**	6.29	-4.09	5.59	13.36	-11.52	23.27	-13.99	7.89	-11.48	0.48	-23.95
**152**	8.04	24.93	15.37	18.04	7.7	15.43	-7.77	-18.24	4.39	-8.55	-14.59
**153**	-16.49	-5.03	-16.12	-3.16	7.49	24.17	4.09	2.67	-2.73	-10.22	-0.65
**154**	-10.98	-5.60	-10.69	12.71	5.3	26.19	7.68	5.30	-2.03	-9.67	-12.10
**155**	5.08	32.30	16.50	5.95	9.85	-7.06	-13.21	-21.20	3.11	8.94	9.10
**156**	12.42	-3.18	11.09	6.79	-10.05	16.82	-13.20	6.74	-11.84	11.27	-23.09
**157**	-11.53	-4.61	-10.16	6.35	5.4	14.5	9.92	3.12	-1.17	-8.77	-0.99
**159**	-14.18	-6.38	-13.05	-13.03	11.48	-36.2	16.03	0.63	-5.86	-18.25	70.40
**160**	-15.14	-2.41	-16.52	-29.24	-10.75	-56.76	-22.79	12.01	-18.87	-	-
**161**	-12.10	-7.33	-11.91	-15.52	-12.49	-87.17	-	-	-	-9.62	35.76
**162**	-14.64	-4.68	-12.65	-3.69	-13.02	-18.4	12.62	0.13	2.43	-10.64	43.18
**163**	-16.50	-4.40	-11.53	13.99	9.23	27.76	10.66	1.75	-0.25	-14.64	-5.94
**164**	-5.81	-0.45	-6.23	19.14	-12.63	26.88	-	-	-	-11.53	-17.07
**165**	-16.99	-7.57	-13.88	0.23	5.56	-2.25	5.99	5.31	0.68	-13.07	26.77
**166**	-17.99	-4.19	-15.74	7.34	-13.13	12.03	-12.87	17.61	11.63	-13.69	6.15
**167**	-14.19	-4.29	-13.19	13.32	-11.06	21.33	-16.64	13.28	-4.16	-12.80	-5.68
**168**	-10.33	-6.41	-11.73	12.89	-12.66	19.14	-10.77	12.65	13.34	-10.41	-8.20
**169**	-15.61	-2.63	-13.24	15.16	-7.59	28.01	-13.20	12.15	-8.45	-14.33	-12.67
**170**	-13.48	-1.59	-12.03	17.79	-7.97	32.5	-3.89	10.07	5.34	-15.40	-23.30
**171**	-15.24	-2.64	-11.95	12.71	-10.73	16.23	-7.03	13.15	6.27	-17.97	-0.79
**172**	-14.39	-2.50	-14.89	14.42	-9.05	33.67	-14.62	13.55	-8.73	-13.35	-26.43
**173**	-12.03	0.68	-10.88	13.62	-11.83	19.92	-11.25	12.81	4.86	-18.96	-8.87
**174**	12.41	-9.62	9.99	-4.51	9.71	-18.52	9.87	0.80	3.86	-4.74	25.71
**175**	-7.48	0.43	-8.08	19.96	-8.08	30.55	4.61	1.46	0.81	-13.02	-23.40
**177**	21.50	-9.50	17.15	-26.26	8.24	-25.61	10.19	-1.01	2.80	24.15	12.80
**178**	-10.72	-3.55	-11.03	14.24	4.77	33.66	7.37	4.22	-0.11	-17.43	-23.85
**179**	-8.99	0.09	-7.91	16.90	8.17	39.19	3.79	3.96	0.57	-8.93	-29.61
**180**	-4.50	-5.14	-8.14	2.28	9.42	22.05	6.84	0.26	-4.08	-3.74	-17.58
**182**	14.68	-7.56	10.78	-39.48	10.15	-62.65	9.41	3.02	4.40	-12.81	73.81
**191**	-10.47	-4.69	-8.56	5.02	-12.51	-6.63	-13.70	11.39	-12.70	-11.41	27.72
**192**	-8.46	-6.77	-9.44	7.60	8.78	10.76	13.19	2.03	3.89	-	-
**193**	-17.01	-5.40	-	-	5.91	-79.49	10.37	-0.50	-3.57	-22.50	80.91
**194**	-19.78	-6.80	-	-	-	-	-10.53	-15.45	-4.21	36.33	73.14
**197**	12.11	-0.75	12.36	-8.03	-9.95	-17.68	4.59	0.53	-0.75	6.64	18.76
**198**	12.83	-4.34	7.73	21.50	-10.96	30.61	-6.23	-8.59	-0.74	-	-
**199**	18.29	-5.17	11.09	5.36	-10	2.13	12.61	1.55	2.35	-	-
**200**	0.99	36.42	17.57	18.50	7.16	12.05	-15.86	-21.90	0.21	-16.19	-1.67

Supplementary table S7 (continued). Score values of the samples.

Sample	Beer style		Fermentation		Purity Law		Grain			Abs. 294nm	
	PC1	PC2	PC1	PC2	PC1	PC2	PC1	PC2	PC3	PC1	PC2
**201**	-11.68	-7.04	-11.96	9.82	5	5.37	8.63	5.08	-1.72	-17.52	17.00
**202**	22.36	-0.21	15.61	15.90	-11.77	-8.14	-12.53	-20.72	4.45	-11.37	8.31
**203**	-14.17	-8.17	-11.16	-7.52	9.89	-9.1	10.40	3.99	1.49	-6.57	30.79
**205**	-13.42	-4.14	-11.62	11.62	3.4	15.3	4.91	5.83	2.46	-13.32	1.99
**206**	-11.35	-5.19	-15.40	-	-11.55	-74.66	5.92	0.71	-2.09	106.76	9.93
**207**	10.61	0.02	15.55	-	-12.81	-92.85	-6.77	-17.18	4.71	73.99	8.55
**208**	-9.40	-5.42	-9.79	8.42	6.77	19.53	10.39	4.06	0.52	-7.46	-8.92
**209**	-2.06	-3.79	-3.57	-8.04	8.68	-8.43	5.74	2.54	2.11	-1.73	15.53
**210**	19.24	-5.80	9.74	-28.22	-11.66	-16.41	13.12	7.44	-1.10	-	-
**211**	18.50	-4.96	16.40	-	10.4	-64.07	13.72	3.06	1.74	93.77	-14.44
**212**	13.53	-8.31	12.19	10.65	7.97	-5.84	4.96	-2.26	1.92	-8.56	21.95
**213**	-9.55	-5.38	-11.78	4.98	-12.22	3.27	-12.88	19.82	17.69	-4.58	13.00
**214**	-5.05	-5.38	-7.89	7.37	8.19	14.19	6.87	2.54	-3.27	-3.96	-1.85
**215**	14.02	-11.11	11.87	-5.64	8.89	-26.37	12.25	1.89	0.52	1.88	42.92
**216**	-11.17	-6.04	-9.75	-18.62	5.57	-22.89	9.76	2.12	-2.14	-6.89	40.03
**217**	8.06	33.98	18.20	0.19	8.26	-7.28	-12.08	-19.90	2.43	4.41	9.45
**218**	17.29	-9.08	14.00	11.63	9.26	3.57	11.81	1.46	-0.90	-6.56	5.20
**219**	17.34	-9.09	13.93	11.60	9	3.52	11.69	1.35	-0.79	-6.70	5.23
**220**	11.38	-8.10	11.22	-2.19	8.25	-16.85	11.00	-0.35	-3.86	1.61	25.86
**221**	18.53	5.13	16.41	0.52	9.58	-14.29	-7.45	-13.45	-1.66	-11.62	21.16
**222**	-8.77	-6.28	-11.56	1.28	-14.44	-3.22	-	-	-	-8.93	16.63
**223**	-10.25	-0.68	-12.37	-44.28	8.75	-28.6	10.33	3.44	-4.12	47.42	20.14
**224**	-9.51	-5.99	-11.14	-4.24	9.14	-2.16	10.64	3.28	1.18	0.37	18.65
**226**	-14.63	-1.73	-13.42	-22.99	8.99	-15.11	8.56	1.12	-4.46	5.77	23.35
**227**	2.00	36.92	15.28	-0.85	9.63	-6.03	-13.81	-20.03	1.48	1.20	10.08
**228**	-13.47	-3.57	-14.38	5.79	-13.42	10.52	-19.75	12.60	-15.16	-10.23	-0.25
**229**	-10.95	-4.50	-13.37	9.35	-12.52	21.41	-18.34	11.72	-12.58	-8.59	-14.96
**230**	-12.65	-4.93	-13.69	11.41	-9.1	19.69	-8.36	9.60	-9.01	-9.09	-10.10
**231**	-15.00	-3.65	-11.91	19.59	-13.19	28.86	-18.73	14.39	-16.69	-7.99	-11.93
**232**	-12.62	-3.65	-13.90	2.92	-10.74	4.77	-	-	-	-0.86	3.08
**235**	-6.74	0.26	-8.36	12.61	-14.36	25.02	-20.27	10.05	-13.60	-	-
**236**	-11.93	-2.31	-12.83	12.06	-12.76	17.02	-24.31	12.45	-17.40	-	-
**237**	-15.15	-3.01	-14.02	8.54	-12.47	13.27	-22.00	13.69	-19.97	-	-
**238**	-18.11	-5.41	-13.60	-79.36	5.93	-56.08	10.52	2.65	-4.97	13.15	57.65
**239**	-11.47	-5.67	-13.34	15.87	-11.18	29.27	-12.59	14.95	2.21	-	-
**241**	-12.90	-2.49	-13.34	14.06	-11.12	29.43	-14.15	15.10	4.54	-	-
**242**	-13.59	-3.96	-12.06	13.22	8.69	24.2	10.23	4.35	-3.22	-14.09	-9.01
**243**	-11.32	-10.21	-9.56	17.38	10.1	14.16	12.32	1.20	-3.64	-9.06	9.63
**244**	-6.87	-3.78	-6.41	8.29	7.18	4.12	9.94	-0.01	-4.07	-15.64	9.88
**245**	-15.11	-2.29	-14.95	14.95	8.21	35.53	6.98	2.75	-1.56	-19.12	-21.47
**246**	-15.19	-4.80	-15.18	10.65	8.95	19.72	11.09	2.66	-1.81	-16.01	-3.42
**247**	-12.30	-1.96	-12.88	11.21	6.94	27.77	4.44	2.31	-2.85	-9.22	-19.27
**248**	-9.03	-2.06	-10.52	19.71	7.73	42.13	7.12	1.09	0.79	-14.95	-34.29
**249**	-9.42	-1.93	-10.81	13.93	7.96	21.91	8.45	2.16	-2.83	-9.58	-10.74
**250**	-1.03	25.17	11.26	21.13	8.41	24.95	-10.91	-15.63	-1.58	-10.93	-17.70
**251**	1.49	21.83	13.35	14.88	6.83	13.02	-4.11	-14.67	0.77	-6.08	-5.75
**252**	-14.04	-3.08	-12.72	14.71	8.98	29.19	11.06	2.68	0.88	-16.04	-13.59
**253**	-1.00	32.68	13.70	20.57	11.1	18.88	-7.29	-21.55	3.42	-13.19	-11.25
**254**	-9.54	-0.13	-9.14	12.32	7.42	20.17	1.65	3.04	1.81	-7.38	-29.57
**255**	-1.52	23.26	9.30	20.15	8.05	29.25	-8.84	-14.53	0.45	-6.64	-26.61
**256**	-3.28	20.25	7.86	25.59	6.68	34.92	-9.50	-11.05	-0.38	-13.28	-28.41
**257**	-3.06	25.55	11.97	30.14	9.28	35.18	-7.11	-18.75	1.59	-12.54	-23.97
**258**	-1.45	26.89	12.73	19.68	9.47	26.56	-6.13	-16.23	3.18	-8.38	-21.51
**259**	3.68	23.03	13.68	8.17	8.4	20.06	-5.89	-15.65	1.40	6.03	-25.07
**260**	-7.18	-2.84	-8.07	12.29	8.7	30.56	6.65	-0.04	2.13	-8.45	-23.79
**261**	-14.05	0.01	-11.26	6.57	9.7	23.62	8.70	1.69	-0.05	-5.96	-15.42
**262**	-14.69	-5.75	-16.19	9.40	7.88	17.16	8.90	3.73	-0.92	-	-
**263**	-5.29	35.14	11.22	18.46	8.87	16.83	-14.09	-19.62	-0.87	-	-
**264**	-13.75	-6.84	-14.89	9.81	-13.25	5.93	-18.04	16.07	-13.07	-	-
**265**	-11.13	-4.97	-13.57	11.38	-13.17	18.26	-18.25	15.63	-7.33	-	-
**266**	-3.39	-1.34	-6.40	19.41	-11.69	36.69	-19.50	10.38	-7.71	-	-
**267**	-11.83	-4.74	-14.08	6.64	-13.77	13.49	-22.09	13.46	-8.36	-	-
**268**	-9.52	-5.15	-11.59	-0.56	-11.86	-3.05	-	-	-	-	-
**269**	-9.18	-0.42	-8.53	8.86	-11.76	12.22	-15.07	7.01	-7.50	-	-
**270**	-7.47	-1.85	-10.36	19.55	-12.37	37.55	-18.38	11.25	-11.27	-	-
**271**	-4.89	1.33	-6.79	22.18	-8.27	55.42	-13.62	8.49	-9.38	-	-
**272**	-3.59	-0.83	-8.10	-1.00	-10.84	6.85	-13.05	14.52	5.69	13.66	-7.59
**273**	23.21	-9.32	15.78	-71.77	10.01	-85.37	9.04	1.41	-0.96	-	-
**274**	-4.67	-0.55	-11.04	-11.45	-12.37	-11.29	-14.59	19.35	13.50	-	-
**275**	-16.02	-3.71	-15.97	10.63	-11.86	20.39	-19.59	17.30	-4.08	-12.94	-8.29
**276**	-13.83	-5.48	-12.55	5.39	-9.04	6.12	-17.75	15.12	-15.38	-	-
**277**	-12.16	-3.39	-12.25	7.23	-10.14	14.28	-13.69	13.55	-9.19	-	-
**278**	23.55	-7.65	17.09	-15.95	7.55	-27.51	4.31	2.80	2.08	-	-
**279**	-4.32	-3.41	-5.99	19.52	6.42	28.42	2.03	5.58	-1.27	-	-
**280**	-12.41	-2.65	-13.26	11.12	-10.13	22.87	-16.13	12.93	-8.09	-	-
**281**	13.17	-5.33	11.90	7.85	6.43	8.59	8.57	1.49	0.65	-	-
**282**	16.47	-3.74	10.60	-32.89	11.51	-22.61	11.96	3.77	-1.13	32.12	-0.53
**283**	-10.94	-5.74	-11.40	9.36	-9.59	16.51	-8.18	11.86	-6.99	-	-
**284**	12.41	-4.99	9.90	11.44	5.7	22.79	6.75	2.50	-0.07	9.90	-30.05
**285**	-9.72	-2.55	-9.68	5.55	-10.11	12.75	-13.94	12.29	-10.30	-3.05	-7.19
**286**	7.87	-6.35	7.10	8.77	7.12	12.11	8.14	2.27	2.19	-	-
**287**	12.31	-4.92	10.21	11.05	10.51	21.5	6.20	0.83	0.18	-	-
**288**	13.58	-4.43	10.05	18.82	-12.13	24.67	2.87	0.95	-0.59	-	-
**289**	7.61	6.38	8.79	24.42	-11.85	29.51	-13.96	-8.64	-2.65	-	-
**290**	8.88	15.73	14.27	13.16	-7.79	11.59	-7.47	-12.76	4.01	1.76	-13.91
**291**	0.52	-2.36	6.39	14.31	-10.43	10.62	4.15	1.00	1.80	-	-

Supplementary table S7 (continued). Score values of the samples.

Sample	Beer style		Fermentation		Purity Law		Grain			Abs. 294nm	
	PC1	PC2	PC1	PC2	PC1	PC2	PC1	PC2	PC3	PC1	PC2
**292**	7.76	-7.16	6.34	17.72	-10.94	10.9	10.22	2.35	1.84	-13.80	-0.77
**293**	-11.21	-1.60	-11.26	-23.09	6.57	-12.61	6.47	3.31	-1.90	12.45	15.12
**294**	-2.88	-8.51	-5.50	1.61	7.92	7.49	6.19	2.17	1.11	-	-
**295**	-6.26	-2.70	-7.18	2.58	9.24	15.35	4.80	1.29	0.87	-	-
**296**	-6.73	-0.03	-7.45	18.93	-13.31	31.44	-16.08	9.57	-6.27	-	-
**297**	-8.54	-2.81	-10.12	14.14	8.06	40.19	-	-	-	-	-
**298**	19.51	-8.88	11.65	-16.89	9.75	-42.06	2.58	1.79	3.13	-	-
**299**	16.77	-2.25	15.40	-10.21	7.84	-19.3	-3.28	-9.13	1.20	-	-
**300**	-8.53	-1.11	-11.69	-48.24	9.53	-24.51	9.72	2.18	-0.03	22.71	13.42
**301**	8.95	-3.78	9.74	0.06	10.04	6.84	13.75	2.65	3.39	-	-
**302**	-7.99	-2.79	-8.92	-5.12	-9.53	-3.28	2.10	5.39	-0.98	-	-
**303**	1.81	40.99	7.94	-20.29	9.62	-36.63	-7.36	-17.79	2.39	-	-
**304**	-11.65	-7.12	-12.11	-18.87	8.48	-35.23	15.52	3.11	-0.48	-	-
**305**	1.16	36.72	14.86	-8.65	7.98	-27.76	-10.36	-18.80	2.24	-	-
**306**	-16.42	-9.54	-14.64	-8.95	7.87	-11.12	9.92	4.81	3.83	-	-
**307**	-4.78	-7.49	-8.06	-6.94	-10.81	-14.48	-6.02	13.42	6.21	-	-
**308**	-17.80	-9.48	24.75	48.75	12.91	9.42	-15.13	-24.82	7.12	-	-
**309**	-13.94	-8.09	-1.55	28.73	-10.09	8.87	-16.87	7.67	18.03	-	-
**310**	15.33	-11.03	15.06	-27.40	9.09	-50.14	8.56	1.59	2.23	-	-
**311**	-7.69	-2.94	-8.18	14.34	-6.08	29.87	-3.01	9.01	2.04	-	-
**312**	-10.34	-4.04	-9.76	9.24	-5.64	21.79	-1.14	8.32	3.64	-	-
**313**	-10.85	-5.10	-11.50	7.15	-9.15	15.7	-5.85	13.48	2.55	-	-
**314**	9.81	30.03	13.26	29.97	-11.28	30.59	-11.41	-22.25	-0.10	-	-
**315**	-3.30	-3.59	9.43	6.67	-13.52	-15.46	11.94	1.34	-2.65	-	-
**317**	-13.70	-6.46	-13.72	4.21	-11.53	5.4	-	-	-	-	-
**318**	-12.80	-7.88	-11.91	7.82	-10.91	5.45	-14.19	19.57	19.00	-	-
**319**	-19.15	-8.17	-16.97	-7.83	-12.3	-8.66	-24.18	17.38	-18.15	-	-
**320**	-9.77	-3.60	-13.27	10.47	-13.9	20.7	-22.83	15.17	-9.88	-	-
**321**	-10.88	-5.28	-12.19	13.59	-8.63	26.02	-7.26	12.50	5.68	-	-
**322**	-14.14	-4.68	-14.42	11.73	-13	21.3	-	-	-	-	-
**323**	-11.49	-8.16	-10.38	-1.76	-12.14	-14.7	-19.29	13.68	-15.29	-	-
**324**	-14.01	-5.77	-14.48	9.52	-13.95	18.38	-19.93	21.34	13.25	-	-
**325**	-8.27	-5.64	-12.41	-0.15	-11.77	15.1	-	-	-	-	-
**326**	-13.82	-0.69	-12.67	18.49	8.31	50.39	4.65	1.47	-3.18	-	-
**327**	-11.07	-4.57	-11.69	7.57	-13.82	15.44	-16.93	19.47	9.50	-	-
**328**	-5.54	-2.75	-9.35	14.94	-12.94	28.59	-24.98	13.26	-10.93	-	-
**329**	-13.56	-4.55	-14.69	9.76	-12.79	11.77	-20.38	14.34	-12.91	-	-
**330**	-14.28	-3.12	-14.13	10.57	-15.14	18.49	-25.39	16.05	-14.58	-	-
**331**	-12.64	-2.74	-13.71	5.99	-12.41	8.42	-10.67	16.73	17.68	-	-
**333**	-15.99	-6.89	-14.35	-1.98	-13.19	19.86	2.03	7.34	-1.75	-	-
**334**	-6.11	-2.71	-10.76	-2.05	-11.4	-18.75	-14.52	18.71	21.59	-	-
**335**	-14.27	-3.37	-13.17	10.23	-10.96	-2.41	-19.21	19.18	24.29	-	-
**336**	-7.59	0.97	-15.12	1.57	-13.91	-24.55	-12.99	14.46	19.36	-	-
**337**	-12.46	-3.52	-13.48	4.00	-9.97	-17.13	-15.92	19.22	23.25	-	-
**357**	19.40	13.52	14.04	13.18	7.42	2.19	-13.40	-19.18	3.30	-	-
**358**	-10.38	-6.24	-12.47	-30.71	9.09	-41.97	8.75	3.20	-0.77	-	-
**360**	17.57	-4.91	9.70	12.76	-13.45	8.46	-	-	-	-	-
**361**	-7.74	-3.55	-12.59	-9.17	-11.01	-26.53	-	-	-	-	-
**362**	-6.42	-2.43	-12.55	4.02	-9.98	-15.61	-	-	-	-	-
**363**	-7.60	0.84	-12.59	12.98	8.49	-19.42	-	-	-	-	-
**364**	18.93	7.41	5.20	-13.77	-7.79	-81.12	-9.00	-8.69	7.60	-	-
**365**	17.17	-5.18	11.96	-40.16	-11.28	-80.56	10.60	1.37	0.56	-	-
**366**	-8.37	-0.76	-13.17	5.40	-12.91	-26.23	-	-	-	-	-
**367**	7.34	35.10	15.51	19.24	8.75	-7.28	-11.53	-17.33	5.13	-	-
**368**	20.00	-7.62	15.50	-22.80	9.27	-96.44	9.30	6.87	-6.00	-	-
**369**	-13.58	-4.79	-13.62	-13.07	9.61	-42.94	9.96	3.89	-0.58	-	-
**370**	16.05	-2.26	10.84	-61.43	10.15	-99.04	13.14	-0.16	0.87	-	-
**371**	0.54	60.45	12.83	-3.56	4.88	-35.26	-7.97	-18.90	3.10	-	-
**372**	1.76	42.19	12.57	3.61	9.71	-28.21	-14.11	-19.18	4.57	-	-
**373**	-16.56	-2.54	-13.74	12.04	-9.83	-1.91	-	-	-	-	-
**374**	-11.72	-2.00	-12.74	14.10	-13.09	7.47	-	-	-	-	-
**375**	-12.69	-4.46	-13.71	0.93	11.13	-17.45	12.13	4.14	-1.52	-	-
**376**	-9.23	-4.56	-12.34	-5.15	8.24	-38.97	8.95	0.28	-7.38	-	-
**377**	-9.50	-3.59	-10.95	17.54	-12.52	20.48	-	-	-	-	-
**378**	-12.93	-2.97	-12.16	11.42	-11.79	5.47	-	-	-	-	-
**379**	-17.78	-7.18	-8.55	4.77	-12.63	-33.09	-24.06	15.98	-21.73	-	-
**380**	19.59	-8.22	13.64	-30.64	7.71	-40.61	14.42	4.03	-0.11	-	-
**381**	-6.82	-4.16	-9.74	8.99	-10.39	22.03	-11.35	14.83	13.09	-	-
**382**	-11.65	-0.65	-11.63	7.21	-9.33	11.53	-9.97	13.37	15.02	-	-
**383**	-12.04	-3.71	-12.66	19.32	-12.57	28.68	-21.36	16.40	-10.11	-	-
**385**	-3.77	-2.55	-15.32	-7.51	7.89	-46.39	17.54	-2.41	-7.86	-	-
**386**	21.40	-8.33	13.68	-13.28	9.97	-15.6	-	-	-	-	-
**387**	19.84	-4.93	12.92	-77.69	10.55	-96.34	13.67	2.95	-2.59	-	-
**388**	-11.05	-4.30	-13.62	16.24	-10.82	11.49	-	-	-	-	-
**389**	19.50	-6.16	14.38	-10.16	-7.55	5.01	10.63	3.91	0.88	49.60	-33.06
**390**	20.13	-5.27	15.77	-27.31	-11.57	-15.49	9.44	4.00	1.33	-	-
**391**	-11.54	-4.68	-13.07	-12.06	-13.62	-22.36	-15.60	16.05	13.59	-	-
**392**	-14.91	-3.05	-11.51	-14.52	9.35	-27.07	12.10	3.02	3.06	-	-
**393**	11.38	-5.59	9.31	17.69	6.46	28.81	6.72	0.34	0.63	-	-
**394**	23.90	-4.10	17.01	-27.68	-14.73	-27.58	-9.00	10.52	9.36	63.23	-4.26
**395**	20.14	-2.30	14.25	17.35	-13.11	-12.34	-12.37	-17.87	-0.90	-	-
**396**	16.41	-6.87	13.23	-3.29	8.97	4.14	9.02	3.26	2.59	-	-
**397**	14.68	-0.10	16.21	42.16	6.48	10.49	-7.40	-16.13	2.27	-	-
**398**	19.33	-6.02	14.37	-6.18	9.31	4.65	9.61	1.26	0.30	31.98	-18.31
**399**	11.87	-2.07	12.39	16.91	9.16	16.11	10.69	-2.68	2.02	-	-

Supplementary table S7 (continued). Score values of the samples.

Sample	Beer style		Fermentation		Purity Law		Grain			Abs. 294nm	
	PC1	PC2	PC1	PC2	PC1	PC2	PC1	PC2	PC3	PC1	PC2
**400**	24.26	3.84	11.05	16.86	-12.14	-9.56	-9.85	-20.28	5.49	-	-
**401**	19.57	-9.41	13.32	-4.03	9.68	-14.44	7.38	-2.79	2.23	-	-
**402**	23.68	-6.70	17.38	6.70	-12.83	-14.12	-9.82	-15.69	2.45	-	-
**404**	21.82	-5.47	15.29	-0.87	-12.72	6.49	-1.33	-7.90	0.53	-	-
**405**	21.51	-10.77	14.24	12.71	-11.03	-5.73	-9.34	-13.19	3.55	-	-
**406**	20.08	-9.04	16.62	16.78	5.98	15.64	8.65	-1.72	2.57	-	-
**407**	-8.81	4.35	-7.30	11.85	-13.21	15.81	-13.66	14.27	13.77	-	-
**408**	-15.83	-4.66	-15.76	-20.48	-13.02	-29.84	-	-	-	-	-
**409**	20.53	-6.37	12.33	-88.67	-11.55	-95.92	-13.37	-23.69	-0.99	-	-
**410**	-18.88	-6.53	-13.92	-15.58	-11.17	-27.15	-13.06	17.89	20.86	-	-
**411**	-9.76	-7.07	-14.21	-18.77	-11.31	-34.72	-25.87	13.57	-18.47	-	-
**412**	-10.82	0.81	-14.32	-19.88	-11.29	-52.56	-12.11	-16.23	4.34	-	-
**413**	-10.02	-7.67	-13.10	-0.28	-14.16	-8.08	-12.62	16.33	17.63	-	-
**414**	-20.22	-5.81	-14.19	-14.99	7.85	-33.57	11.63	2.17	-1.84	-	-
**416**	12.86	-0.48	13.39	-1.56	10.28	1.68	9.41	-1.47	-0.23	13.97	-5.52
**417**	21.73	-5.58	15.92	-14.67	6.45	-0.69	7.80	1.33	1.51	48.73	-24.17
**418**	18.50	-6.55	14.19	-33.78	8.27	-12.36	12.42	4.72	0.82	46.64	-13.90
**419**	11.19	-2.76	8.51	-2.18	6.6	16.61	8.57	4.09	1.11	31.06	-30.57
**420**	19.82	-6.39	14.13	-28.94	-11.67	-36.26	10.35	2.25	-2.12	29.20	16.49
**421**	16.57	-4.95	10.76	3.05	8.06	32.48	7.86	-0.69	-2.28	27.16	-33.86
**422**	14.01	0.17	13.02	-31.53	-11.41	-32.06	6.91	1.13	1.47	51.61	10.79
**424**	26.03	-7.54	15.71	-65.94	-11.14	-49.78	6.87	1.49	4.28	71.43	-4.75
**425**	19.30	-6.36	13.52	-57.59	-11.67	-41.25	6.28	1.54	1.85	32.73	12.70
**426**	21.94	-13.26	-	-	-	-	19.33	-0.64	-3.71	-	-
**427**	12.57	-2.88	9.86	-10.87	7.4	6.92	9.24	3.69	0.75	50.94	-28.36
**428**	8.85	-3.31	8.44	16.64	7.41	30.09	8.73	0.75	1.76	4.23	-32.21
**429**	-9.42	-4.42	-9.70	8.20	3.85	18.16	5.78	5.34	-0.68	-6.53	-7.96
**430**	17.03	-4.63	13.13	12.82	5.27	32.86	9.81	1.43	0.33	23.26	-48.90
**431**	10.87	-0.64	9.87	0.92	-14.09	18.42	4.95	1.52	-0.15	29.76	-29.98
**432**	21.71	-8.60	15.29	-73.04	8.91	-56.37	9.38	1.65	-0.95	67.59	10.76
**433**	-12.92	-4.28	-14.09	-40.52	7.48	-56.75	16.87	6.85	-5.87	-	-
**434**	-14.03	-11.99	-13.64	-3.34	7.67	-18.25	14.37	3.77	3.53	-	-
**435**	-8.14	-10.48	-12.58	1.76	7.62	0.52	10.42	-1.48	1.29	-	-
**436**	-7.07	-11.17	-9.78	0.77	8.04	-1.01	10.74	-0.94	1.62	-	-
**437**	27.92	-8.77	18.48	17.04	-12.6	-19.78	-12.37	17.13	18.95	-	-
**438**	23.77	-16.78	18.63	37.91	7.2	-14.95	12.49	4.63	-3.96	-	-
**439**	15.22	1.62	14.23	23.87	-6.42	20.45	-1.64	-11.50	0.67	-	-
**440**	15.03	-3.66	10.03	-43.85	9.72	-41.63	7.11	2.96	-4.13	-	-
**441**	18.43	1.30	11.23	-11.80	-10.93	-29.95	-14.15	-21.57	2.26	-	-
**442**	6.24	18.99	15.13	22.81	-8.68	16.82	-11.55	-14.12	-0.68	-	-
**443**	-1.78	-5.44	-6.28	-30.63	6.94	-23.53	8.27	2.67	-2.84	-	-
**444**	-2.00	-3.43	-6.68	10.65	10.53	31.22	5.46	-1.93	-1.57	-	-
**445**	29.00	-9.42	20.25	-27.16	9.27	-75.19	13.72	4.17	-5.14	-	-
**446**	21.44	-4.15	9.89	-70.99	-10.2	-83.34	-9.56	-13.93	-1.58	-	-
**447**	16.64	-7.29	12.08	2.11	8.15	-6.59	9.14	1.58	1.14	-	-
**448**	18.60	-7.28	15.73	5.97	9.17	-100.2	7.70	0.46	-0.44	-	-
**449**	11.27	6.46	12.54	31.45	-9.29	12.08	-6.07	-13.26	3.26	-	-
**450**	-7.05	5.12	-8.90	3.44	7.73	-7.3	-8.42	-15.81	3.65	-	-
**451**	27.53	-1.93	14.19	-9.90	-11.37	-6.76	7.74	5.49	-3.52	-	-
**452**	9.55	-3.80	6.70	24.23	-12.16	19.01	-8.67	9.82	13.53	-	-
**453**	3.63	24.35	14.72	17.80	9.9	20.92	-6.69	-16.87	5.69	-	-
**454**	10.83	-2.05	8.81	13.28	-13.82	12.2	6.08	3.42	2.69	-	-
**455**	-1.54	-5.20	-5.76	-4.39	-9.9	6.25	10.54	8.25	3.55	-	-
**456**	18.37	-3.45	14.68	9.91	-12.35	9.89	5.42	2.35	0.10	-	-
**457**	-13.03	-3.52	-11.92	-16.90	9.68	-19.33	13.21	1.41	2.34	-	-
**458**	-11.68	-4.61	-12.98	-31.08	8.98	-27.63	7.66	0.81	2.74	-	-
**459**	-14.86	-7.19	-13.27	-5.11	7.5	-5.26	9.85	6.32	2.27	-	-
**460**	-13.73	-4.25	-11.45	15.76	7.38	25.43	-	-	-	-	-
**464**	-15.30	-2.52	-12.67	12.67	-10.15	22.77	-8.90	14.38	14.69	-	-
**465**	-7.41	-2.32	-9.22	18.33	-12.76	37.03	-5.39	13.22	9.88	-	-
**467**	-16.88	-6.17	-15.94	-1.55	-8.72	5.29	-5.40	14.15	13.61	-	-
**B1885**	-9.28	2.74	-5.54	10.89	3.82	12.14	-14.17	-2.56	4.19	-16.69	20.60
**B2019**	-12.41	-1.52	-10.01	14.94	5.38	20.19	8.57	6.63	0.29	-18.08	3.41

Supplementary figures

A **(I)**

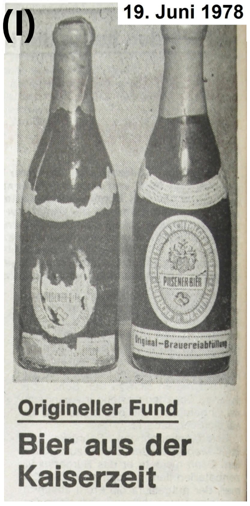

(II)

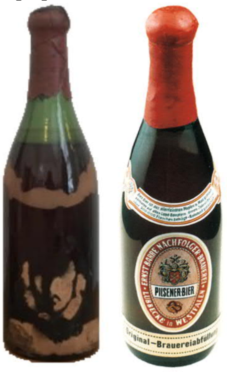

(III)

Newspaper article (19th June 1978): An original find. Beer from the German Empire era.

Beer label:
This beer is brewed from the very finest hops and malt and excellent brewing water, represented in all Lloyd-steamers, whose annual purchase is over 300,000 bottles. Purity guaranteed. Ernst Barre successor brewery, Lübbecke in Westphalia. Original brewery filling.

B

(I)

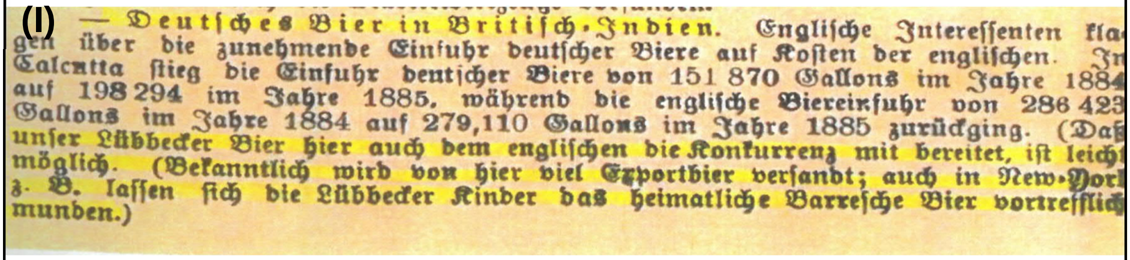

— Deutsches Bier in Britisch-Indien. Englische Interessenten kla-gen über die zunehmende Einfuhr deutscher Biere auf Kosten der englischen. In Calcutta stieg die Einfuhr deutscher Biere von 151 870 Gallons im Jahre 1884 auf 198 294 im Jahre 1885, während die englische Biereinfuhr von 286 423 Gallons im Jahre 1884 auf 279,110 Gallons im Jahre 1885 zurückging. (Daß unser Lübbecker Bier hier auch dem englischen die Konkurrenz mit bereitet, ist leicht möglich. (Bekanntlich wird von hier viel Exportbier versandt; auch in New-York z. B. lassen sich die Lübbecker Kinder das heimatliche Barresche Bier vortrefflich munden.)

(II)

German beer in British India.
English interests complain of increasing imports of German beers at the expense of English ones. In Kolkata, imports of German beers increased from 151,870 gallons in 1884 to 198,294 in 1885, while English beer imports decreased from 286,423 gallons in 1884 to 279,110 gallons in 1885. It is quite possible that the Lübbecker beer here is now competing with the English beer. (As is well known, a lot of export beer is shipped from here; even in New York, for example, the children of Lübbecke enjoy the local Barre beer).

Supplementary Figure S1. Historical sources about the discovery and origin of the beer. A Newspaper article of June 1978 (**A-I**) describes the finding of the unique sample from the German Empire era. The historical beer sample and a replica are shown in **A-II**. The German heading of the newspaper article and the beer's label are translated in **A-III**. Another article refers to the export of Barre beers beginning with the year 1885 and the resulting new competition on the world market (**B-I**). It is translated in **B-II**. The pictures of the beer bottles, the newspaper article and the historical article are used under explicit permission of Privatbrauerei Ernst Barre GmbH.

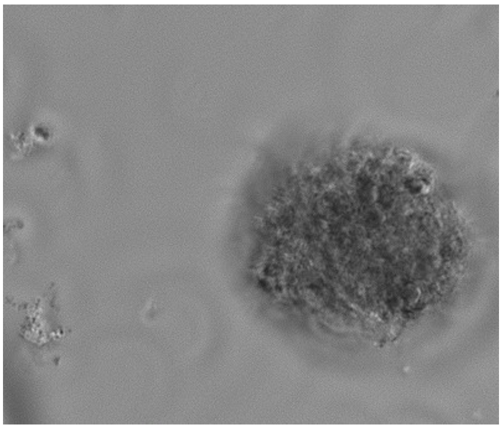

Supplementary Figure S2. Phase-contrast microscopy of amorphous organic particles of 1885 beer. Typical particles that occur during the aging of filtered beers due to polyphenol-protein complexation.

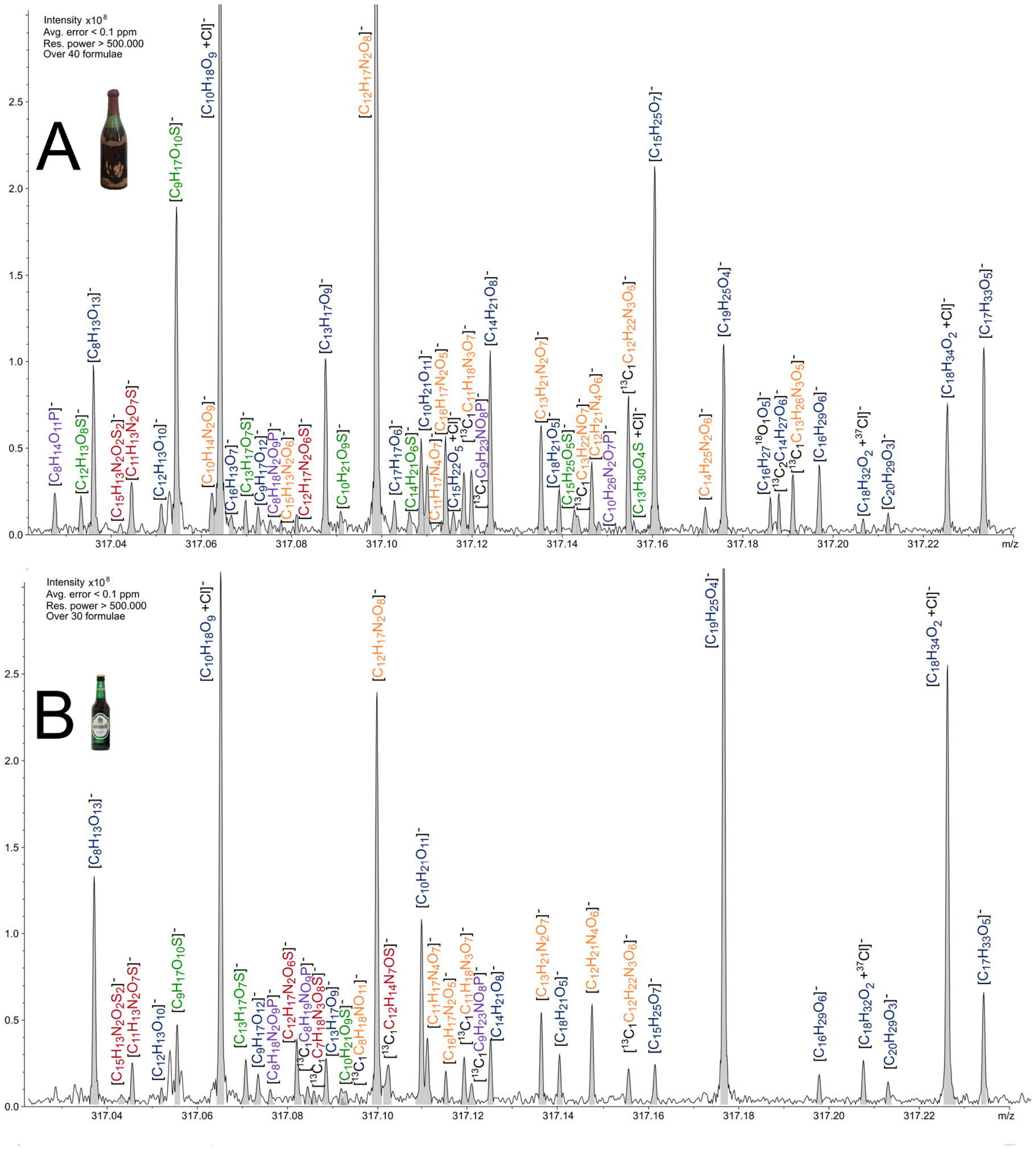

Supplementary Figure S3. FTICR mass spectrum excerpt of beer B1885 (A) and B2019 (B) showing over 40 different compositions in the nominal mass m/z 317. Chemical space color code: CHO (blue), CHNO (orange), CHOS (green), CHNOS (red), CH(N)O(S)P (violet). The pictures of the beer bottles are used under explicit permission of Privatbrauerei Ernst Barre GmbH.

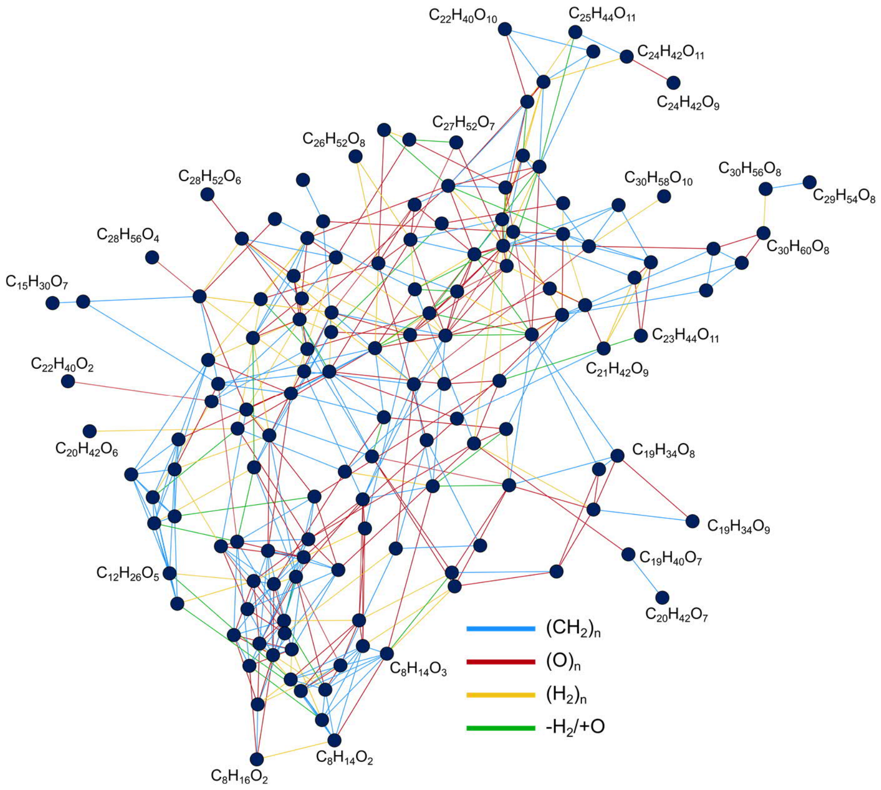

Supplementary Figure S4. Mass difference network of specific lipid-type compositions (as specified by their compositional area in the van Krevelen diagram) for the historical beer. The compositions are represented as nodes that are connected by edges, representing changes in the molecular formula equivalent to (bio-)chemical redox processes.

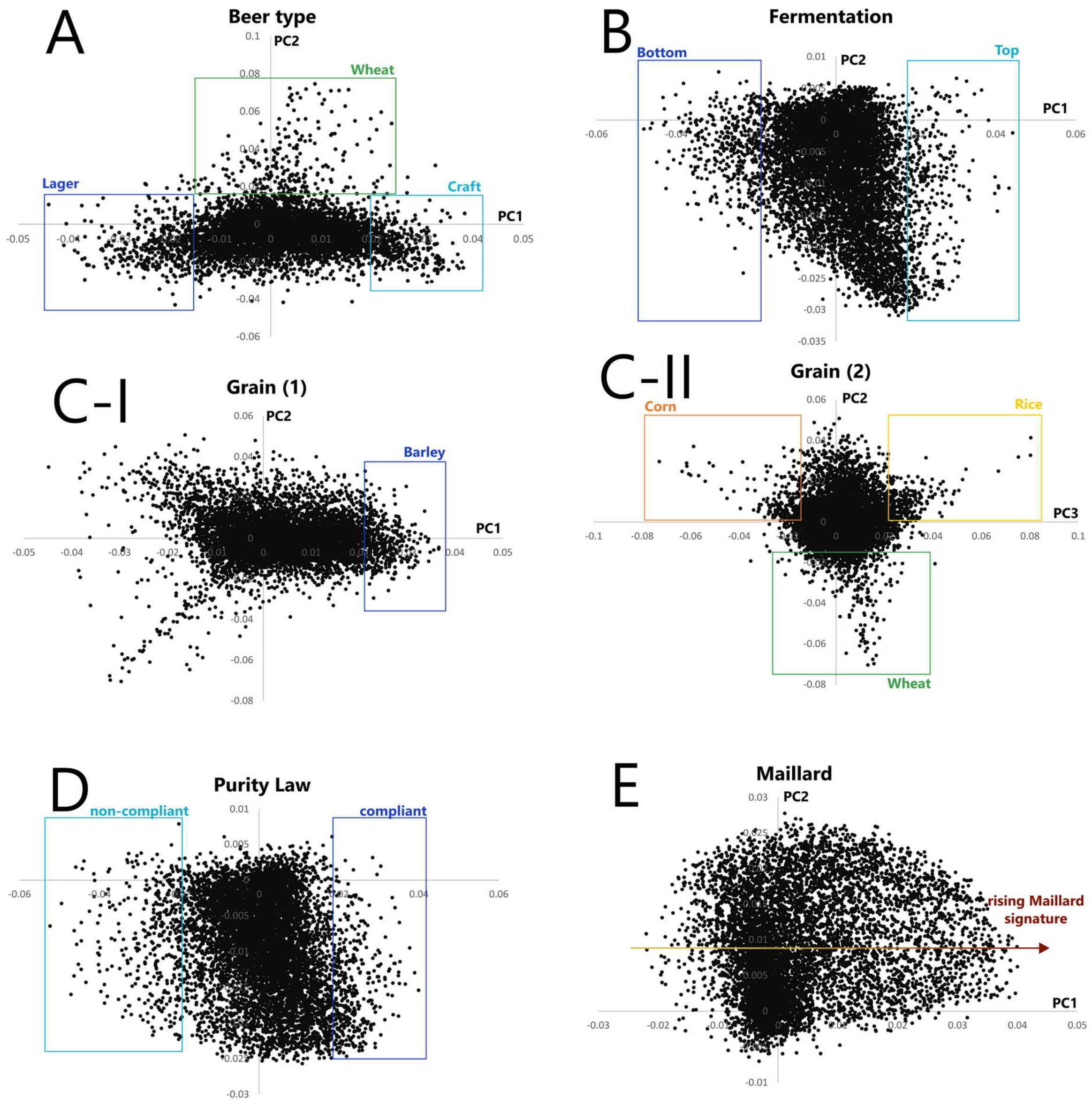

Supplementary Figure S5. Loading plots including 7,700 compositions for the OPLS-DA differentiating beer types (A), fermentation types (B), grains used (C), compliance with the German purity Law (D) and Maillard signatures by the absorption at 294 nm (E). The features specific for an attribute (unique or significantly more abundant) are highlighted. The 95th percentile is highlighted.

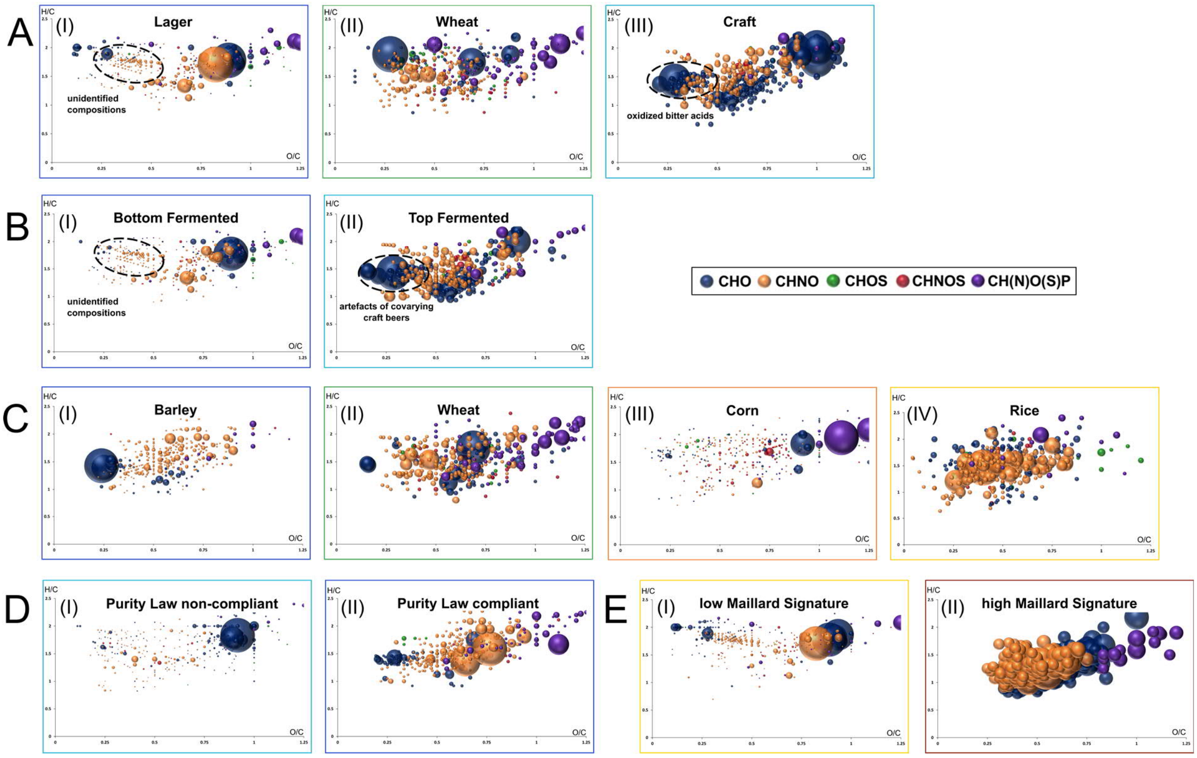

Supplementary Figure S6. Van Krevelen diagrams of the characteristic compositional profiles for beer types (A), fermentation types (B), grains used (C), compliance with the German Purity Law (D) and Maillard signatures (E). Color code: CHO (blue), CHNO (orange), CHOS (green), CHNOS (red), CH(N)O(S)P (violet). Neutral compositions are depicted. Specific areas are highlighted. Bubble size indicates relative signal intensities.

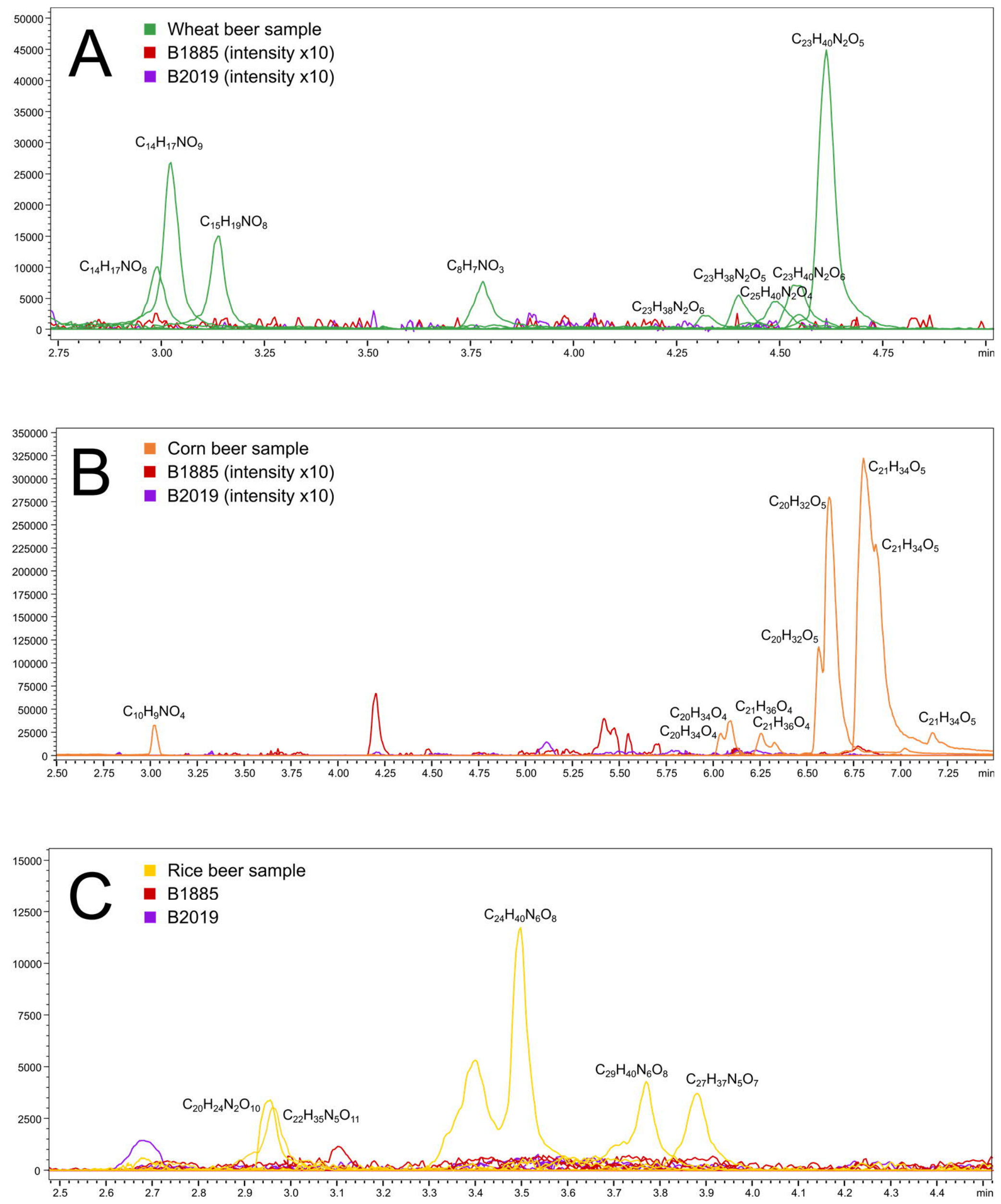

Supplementary Figure S7. Extracted Ion chromatograms of compound masses found to be specific for wheat (A), corn (B) and rice (C) including a respective grain-containing sample, sample B1885 and sample B2019. The EIC mass range is the respective exact *m/z* –value ± 10 ppm**.** No overlap of reported marker molecules (Pieczonka et al. (2021), Front. Chem., 9 (715372), 1-12) between the grain-containing samples and samples B1885 and B2019 was observed.
